# Monitoring maternal near miss/severe maternal morbidity: A systematic review of global practices

**DOI:** 10.1371/journal.pone.0233697

**Published:** 2020-05-29

**Authors:** Natalie England, Julia Madill, Amy Metcalfe, Laura Magee, Stephanie Cooper, Charleen Salmon, Kamala Adhikari

**Affiliations:** 1 Department of Medicine, Cumming School of Medicine, University of Calgary, Calgary, Canada; 2 Department of Obstetrics and Gynaecology, Cumming School of Medicine, University of Calgary, Calgary, Canada; 3 Department of Community Health Sciences, Cumming School of Medicine, University of Calgary, Calgary, Canada; 4 King’s College London, London, United Kingdom; 5 University of Limerick, Limerick, Ireland; University of South Florida, UNITED STATES

## Abstract

There is international interest in monitoring severe events in the obstetrical population, commonly referred to as maternal near miss or severe maternal morbidity. These events can have significant consequences for individuals in this population and further study can inform practices to reduce both maternal morbidity and mortality. Numerous surveillance systems exist but we lack a standardized approach. Given the current inconsistencies and the importance in monitoring these events, this study aimed to identify and compare commonly used surveillance methods. In June 2018, we systematically searched MEDLINE, EMBASE, and CINAHL using terms related to monitoring/surveillance and maternal near miss/severe maternal morbidity. We included papers that used at least three indicators to monitor for these events and collected data on specific surveillance methods. We calculated the rate of maternal near miss/severe maternal morbidity in hospitalization data obtained from the 2016 US National Inpatient Sample using five common surveillance methods. Of 18,832 abstracts, 178 papers were included in our review. 198 indicators were used in studies included in our review; 71.2% (n = 141) of these were used in <10% of included studies and only 6.1% (n = 12) were used in >50% of studies included in our review. Eclampsia was the only indicator that was assessed in >80% of included studies. The rate of these events in American hospitalization data varied depending on the criteria used, ranging from 5.07% (95% CI = 5.02, 5.11) with the Centers for Disease Control criteria and 7.85% (95% CI = 7.79, 7.91) using the Canadian Perinatal Surveillance System. Our review highlights inconsistencies in monitoring practices within and between developed and developing countries. Given the wide variation in monitoring approaches observed and the likely contributing factors for these differences, it may be more feasible for clinical and academic efforts to focus on standardizing approaches in developed and developing countries independently at this time.

**PROSPERO Registration**: CRD42018096858.

## Introduction

Maternal morbidity refers to any health condition attributed to and/or aggravated by pregnancy and childbirth that has a negative impact on the woman’s wellbeing [1–3]. Maternal morbidity is a broad concept; it can include less severe items such as nausea or pruritus in pregnancy as well as more severe or life-threatening events such as eclampsia or postpartum haemorrhage. These cases can have significant short-term and long-term impacts on a woman and her family, including negative impacts on her physical, mental, and/or sexual health, her mobility, her ability to work and engage in other activities in society, and her socioeconomic status [1–6]. In addition to these serious effects on women and their families, high rates of maternal morbidity can contribute to increased financial burden on the healthcare system [4]. Surveillance methods for severe cases of maternal morbidity are inconsistent at present, and as such, estimates on the rate of these events vary. The World Health Organization (WHO) completed a systematic review of the prevalence of severe cases of maternal morbidity in 2004 which showed that the reported rate of these events differed based on the monitoring criteria used [5]. In developed countries, they found that 0.4% of women delivering in hospitals experienced severe acute maternal morbidity (SAMM) when organ-system based criteria were used, however this rate increased to 1% when disease-specific criteria were used for monitoring practices [5]. Similarly, in developing countries, the rate of women delivering in hospitals who experienced severe acute maternal morbidity was 1% when organ failure criteria were used and 4–8% using case-identification criteria [5]. More recently, the rate of severe cases of maternal morbidity has been reported as occurring in 1.42% of Canadian deliveries in 2014/2015 and 1.29% of deliveries in the United States in 2008/2009 [3, 4].

The WHO Working Group on Maternal Mortality and Morbidity Classifications released initial surveillance recommendations on maternal near miss in 2009 [6]. These recommendations were further updated in 2011 [7]. The WHO working group on this topic has advocated for the use of the term maternal near miss (MNM) as opposed to other terms such as severe maternal morbidity (SMM) as they believe that this term best reflects the severity of these events/conditions [6]. They define a maternal near miss case as “a woman who nearly died but survived a complication that occurred during pregnancy, childbirth or within 42 days of termination of pregnancy” [7]. It is also stated that this can be practically thought of as a case in which a woman survives a life-threatening condition (i.e. organ dysfunction) [7]. This method includes clinically observed indicators relating to severe maternal complications such as severe postpartum haemorrhage and severe pre-eclampsia, critical interventions such as admission to an intensive care unit and use of interventional radiology procedures, and organ dysfunction using a number of organ-specific clinical and laboratory criteria [7].

The Centers for Disease Control (CDC) in the United States and the Canadian Perinatal Surveillance System (CPSS) use the term SMM, as opposed to MNM, to describe these events in the maternal population. Both organizations have used recent research on SMM to inform the development of lists of International Classification of Diseases (ICD) diagnosis and procedure codes for surveillance of these events; however, monitoring practices in these countries remain inconsistent [3, 8–10]. The United Kingdom approach to surveillance on maternal morbidity uses a changing list of events and conditions which are seen as priorities for maternal health; they do not have a standard list of indicators of severe events in the maternal population [11]. These events are usually referred to as maternal near miss events in the United Kingdom.

Numerous other methods are also currently in use. The Maternal Morbidity Outcome Indicator (MMOI) published by Roberts et al. in 2008 is another example of a surveillance method using ICD diagnosis and procedure codes; this method was developed and validated using routinely collected health data for women delivering in the Australian state of New South Wales [12]. Mantel et al. (1998) proposed clinical criteria for maternal near miss events using organ system- and management-based criteria based on their research using data from a number of centres in South Africa [13]. Waterstone et al. developed their method, published in 2001, using data on the maternal population within a region of the United Kingdom [14]. The Waterstone et al. definition of severe obstetric morbidity (SOM) includes a number of diagnoses/conditions with specific definitions and intentionally excludes management-based criteria and diagnoses/conditions that are difficult to accurately diagnose [14].

As all of the above approaches use inconsistent methods to monitor MNM/SMM, no reliable comparisons can be made within and between these countries. This limits our ability to truly understand these events, and to develop and implement strategies to reduce the occurrence of MNM/SMM. Additionally, it is unclear whether the variation in reported rates of MNM/SMM is merely a reflection of the differences in monitoring and reporting these events, or if this variation is due to different practices or patient populations leading to different outcomes. Given the incidence of these events and the seriousness of the potential consequences for individuals in the maternal population, their families and communities, and the societal/financial implications for healthcare systems, the importance of studying this concept is evident. Reaching a consensus on terminology and indicators is an important first step in addressing this issue. Thus, this systematic review aimed to assess and summarize what terms and indicators are currently being used to monitor MNM/SMM.

## Methods

A systematic search of three online databases (MEDLINE, EMBASE, and CINAHL) was conducted to identify all relevant studies published prior to June 7, 2018 inclusive. The search strategy was developed by consulting experts in the field and a research librarian who has expertise in systematic review searching. The search involved combining terms related to MNM/SMM and terms related to monitoring to identify relevant papers for inclusion (see S1 Appendix). There were no language restrictions. Our review was prospectively registered in PROSPERO (PROSPERO Registration: CRD42018096858).

After removal of duplicate citations using Covidence, two teams of two reviewers each (NE & KA, AM & CS) independently screened the titles and abstracts of the identified studies. Studies were included in the review if they assessed MNM/SMM during pregnancy, childbirth, and/or up to 42 days postpartum and used at least three distinct indicators of MNM/SMM. This criterion was used to ensure that we were capturing studies that assessed MNM/SMM as a concept and not individual events/conditions in the maternal population. Additionally, at least one of the indicators had to be similar to an indicator used in the WHO near-miss approach to ensure that we were capturing data on severe events [7]. There were no restrictions on study location. Studies that did not meet the above criteria were excluded. Review papers, conference abstracts, case reports, and case series were also excluded. We initially did not exclude secondary analyses of the same dataset/study, but during our data extraction, concerns were raised regarding potential inaccurate weighting of indicator use with the inclusion of multiple secondary analyses from large prospective cohort studies, thus we chose to exclude them at this stage. If the original study was not included in our review, we kept one secondary analysis to ensure that the indicators used would be included once. All secondary analyses of large administrative datasets were included. Disagreement in potential inclusion of a study at any stage was resolved by discussion between the reviewers.

A structured data collection tool developed by our team was used for data extraction (see S2 Appendix). Some studies evaluated maternal death as an indicator of MNM/SMM; given that our review focuses on maternal morbidity and not mortality, we did not collect data on which studies included maternal death.

Due to the variation in design/methods of the studies included in our review, we were unable to find an appropriate standardized quality assessment tool. However, we used a scale to categorize included papers based on method of indicator selection as a surrogate marker for quality. A score of 0 was given if no information was provided on how included indicators were chosen, 1 if the indicators were chosen by the authors of the study and the methods were described, 2 if the indicators were used in previous studies, and 3 if the WHO or CDC methods were used [7, 8].

We analyzed trends in terms and indicators used, methods of indicator selection, admission type (i.e., antepartum admissions, delivery admissions, postpartum admissions, or a combination of the three), and uptake of the WHO near-miss approach. To demonstrate the potential impact that different definitions of MNM/SMM can have on the incidence of MNM/SMM, we used five common approaches (CDC, CPSS, MMOI, Mantel et al., and Waterstone et al.) to calculate the rate of MNM/SMM on hospitalization data obtained from the 2016 US National Inpatient Sample (NIS) database [8, 9, 12–17]. The NIS database is a large publicly available all-payer inpatient care database in the US which contains data from more than 7 million hospital stays each year. The database was designed to produce the estimates of inpatient utilization, access, cost, quality, and outcomes to inform decision making. The database was developed by the Healthcare Cost and Utilization Project through a Federal-State-Industry Partnership sponsored by the Agency for Healthcare, Research, and Quality. The NIS database can be accessed at https://www.hcup-us.ahrq.gov/db/nation/nis/nisdbdocumentation.jsp. We used International Classification of Diseases, Tenth Revision, Clinical Modification (ICD-10-CM) diagnostic codes and ICD-10 procedure codes provided by the authors for the CDC, CPSS, and MMOI lists of indicators, and used the ICD codes developed by Sousa et al. for the Mantel et al. and Waterstone et al. criteria to identify MNM/SMM in the NIS database (see S3 Appendix) [8, 9, 12–17]. All diagnostic and procedure codes, except for the CDC, were identified outside of the US, therefore, each diagnostic and procedure code and associated MNM/SMM indicator were cross-checked with the ICD-10-CM diagnostic codes and ICD-10 procedure codes to ensure accurate representation of the MNM/SMM indicators as proposed by each author. We were unable to calculate the rate of MNM/SMM using the WHO critiera. Some of the WHO criteria are based on specific clinical or laboratory markers (such as hypoperfusion or lactate >5mmol/l, severe acute thrombocytopenia defined as <50000 platelets/ml, severe bradypnea defined as respiratory rate <6 breaths per minute, etc.); these are not possible to identify in the NIS data as this data only contains diagnoses and procedures. Furthermore, we could not find proposed ICD codes for the WHO criteria. The rates of MNM/SMM and 95% confidence intervals were estimated using each of these definitions of MNM/SMM [8, 9, 12–14]. As all data used for this study was publicly available, this study was deemed exempt from review by the Conjoint Health Research Ethics Board at the University of Calgary.

## Results

**Fig 1:**
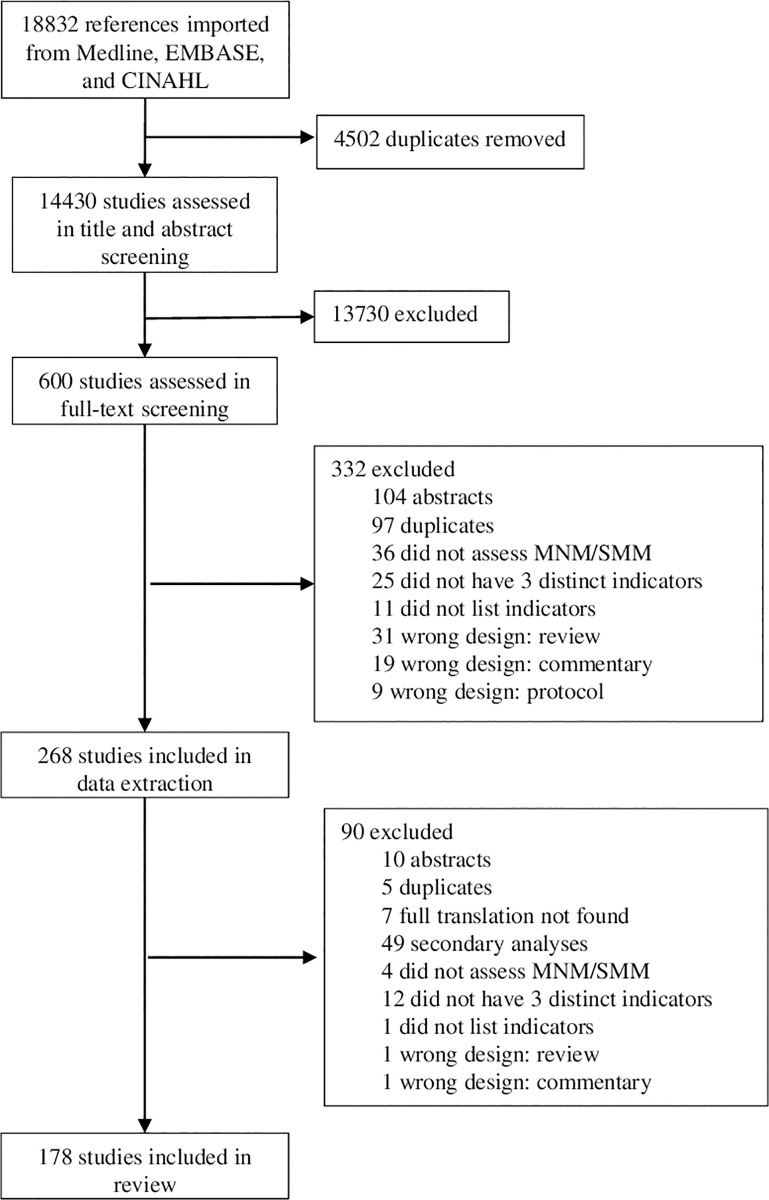
PRISMA flow diagram for systematic review of global monitoring practices for maternal near miss/severe maternal morbidity.

Our initial search yielded 18,832 articles, of which 178 were included in the review. Fig 1 provides a PRISMA flow chart outlining the phases of the systematic review. S4 Appendix provides detailed information on all included papers.

### Terminology

**Fig 2:**
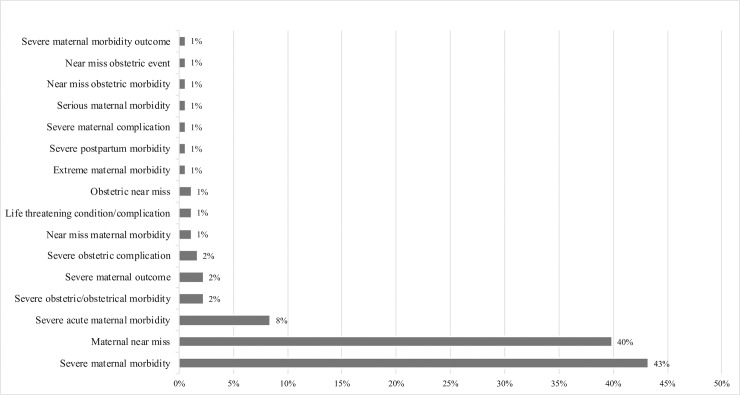
Frequency of term use for included studies.

Of the 178 included papers, 43.3% (n = 77) used the term SMM, 39.9% (n = 71) used MNM, and 21.9% (n = 39) used a different term (nine papers used two terms and were counted twice in these calculations). Of the other terms used, severe acute maternal morbidity (SAMM) was most common (8.4%, n = 15), followed by severe obstetric morbidity (SOM) (2.3%, n = 4), severe maternal outcome (SMO) (2.3%, n = 4), and severe obstetric complications (SOC) (1.69%, n = 3) (Fig 2). Prior to the WHO Working Group on Maternal Mortality and Morbidity Classifications’ 2009 recommendation to use the term MNM, 11.1% (n = 3) of included papers used this term, while 45.0% (n = 68) of studies have used MNM since 2009 [6].

### Indicators

In total, there were 198 unique indicators assessed in studies included in our review. It is important to note that the frequencies reported represent data extracted as written by authors, thus “pre-eclampsia” and “pre-eclampsia with jaundice” are calculated separately, as are the different types of haemorrhage, and any indicators that use diagnoses/clinical conditions linked with management such as “postpartum haemorrhage” and “postpartum haemorrhage requiring transfusion”. Out of the 198 indicators, 71.2% (n = 141) were used in <10% of included studies and only 6.1% (n = 12) were used in >50% of studies included in our review. These 12 include eclampsia (81.5%, n = 145); transfusion (70.8%, n = 126); cerebrovascular disorders (69.7%, n = 124); hysterectomy (69.1%, n = 123); sepsis or septicaemia (67.4%, n = 120); ventilation (67.4%, n = 120); cardiac arrest (62.9%, n = 112); uterine rupture (57.3%, n = 102); ICU (or equivalent) admission (55.1%, n = 98); shock–general or unspecified (53.9%, n = 96); coma, prolonged unconsciousness, or loss of consciousness (51.7%, n = 92); and pre-eclampsia (51.1%, n = 91). Eclampsia was the only indicator that was included in >80% of studies. Of the 12 indicators included in >50% of all studies included in our review, the WHO near-miss approach includes indicators similar to all twelve, the CPSS criteria includes ten, the CDC criteria includes eight, the MMOI criteria includes eight, Mantel et al.’s criteria includes nine, and Waterstone et al.’s criteria includes five. Table 1 provides detailed information regarding common MNM/SMM surveillance methods and their use of indicators related to those assessed in >50% of included studies as well as the inconsistencies in language used for each indicator [8, 9, 12–14].

**Table 1:** Common MNM/SMM surveillance methods and their use of indicators related to those assessed in >50% of included studies.

Indicators assessed in ≥50% of studies	WHO near-miss approach^a^	CPSS^b^	CDC^c^	MMOI^d^	Mantel et al.^e^	Waterstone et al.^f^
**Clinical complications**						
Eclampsia	**+**	**+**	**+**			**+**
Cerebro-vascular disorders	**+**	**+**	**+**	**+**	**+**	
Sepsis or septicaemia	**+**	**+**	**+**		**+**	**+**
Cardiac arrest	**+**	**+**	**+**	**+**	**+**	
Uterine rupture	**+**	**+**		**+**		**+**
Shock–general or unspecified	**+**	**+**	**+**	**+**		
Coma, prolonged unconsciousness, or loss of consciousness	**+**	**+**		**+**	**+**	
Pre-eclampsia	**+**				**+**	**+**
**Interventions**						
Transfusion	**+**	**+**	**+**	**+**	**+**	**+**
Hysterectomy	**+**	**+**	**+**	**+**	**+**	
Ventilation	**+**	**+**	**+**	**+**	**+**	
ICU (or equivalent) admission	**+**				**+**	

^a^ Differences in WHO terminology regarding specific indicators:

- “Stroke” instead of “cerebrovascular disorders”

- In addition to “sepsis”, includes “severe systemic infection”

- “Severe pre-eclampsia” instead of “pre-eclampsia”

- “Use of blood products” instead of “transfusion”

- “Uterine haemorrhage or infection leading to hysterectomy” instead of “hysterectomy”

- “Intubation and ventilation not related to anaesthesia” instead of “ventilation”

^b^ Differences in CPSS terminology regarding specific indicators:

- “Cerebral venous thrombosis in pregnancy”, “cerebral venous thrombosis in the puerperium”, “cerebrovascular diseases: subarachnoid and intracranial haemorrhage, cerebral infarction, stroke” instead of “cerebrovascular disorders”

- “Septicaemia during labour”, “Puerperal sepsis” instead of “sepsis” or “septicaemia”

- “Rupture of uterus before onset of labour”, “Rupture of uterus during labour” instead of “uterine rupture”

- “Obstetric shock” instead of “shock–general or unspecified”

- “Blood transfusion (whole blood or red cell transfusion)”, “placenta previa with haemorrhage + blood transfusion”, “intrapartum haemorrhage + blood transfusion”, postpartum haemorrhage + blood transfusion” instead of “transfusion”

- “Caesarean hysterectomy”, “Total hysterectomy, open approach”, “Subtotal hysterectomy, open approach”, “Postpartum haemorrhage + hysterectomy” instead of “hysterectomy”

- “Assisted ventilation through endotracheal tube”, “Assisted ventilation through tracheostomy” instead of “ventilation”

^c^ Differences in CDC terminology regarding specific indicators:

- “Cardiac arrest/ventricular fibrillation”, “heart failure/arrest during surgery or procedure” instead of cardiac arrest

^d^ Differences in MMOI terminology regarding specific indicators:

- “Cerebro-vascular accident” instead of cerebrovascular disorders

- In addition to “uterine rupture”, includes “repair ruptured or inverted uterus”

- “Transfusion of blood or coagulation factors” instead of “blood transfusion”

- “Assisted ventilation including tracheostomy” instead of “ventilation”

^e^ Differences in Mantel et al. terminology regarding specific indicators:

- “Subarachnoid or intracerebral haemorrhage” instead of “cerebrovascular disorders”

- “Intensive care admission for sepsis”, “emergency hysterectomy for sepsis” instead of “sepsis”

- “Coma in a patient lasting >12 h.” instead of “coma”

- “Jaundice in the presence of preeclampsia” instead of “preeclampsia”

- “Hypovolaemia requiring ≥5 units whole blood or packed cells for resuscitation”, “Acute thrombocytopenia requiring a platelet transfusion” instead of “transfusion”

- “Emergency hysterectomy for any reason”, “Emergency hysterectomy for sepsis” instead of “hysterectomy”

- “Intubation and ventilation for more than 60 minutes for any reason other than for a general anesthetic” instead of “ventilation”

- In addition to “intensive care admission for any reason”, includes “intensive care admission for sepsis”

^f^ Differences in Waterstone et al. terminology regarding specific indicators:

- “Severe sepsis” instead of “sepsis”

- “Severe pre-eclampsia” instead of “pre-eclampsia”

- “Acute transfusion of 4 or more units of blood” is included under their definition of “severe haemorrhage” instead of “transfusion”

### WHO near-miss approach

**Fig 3:**
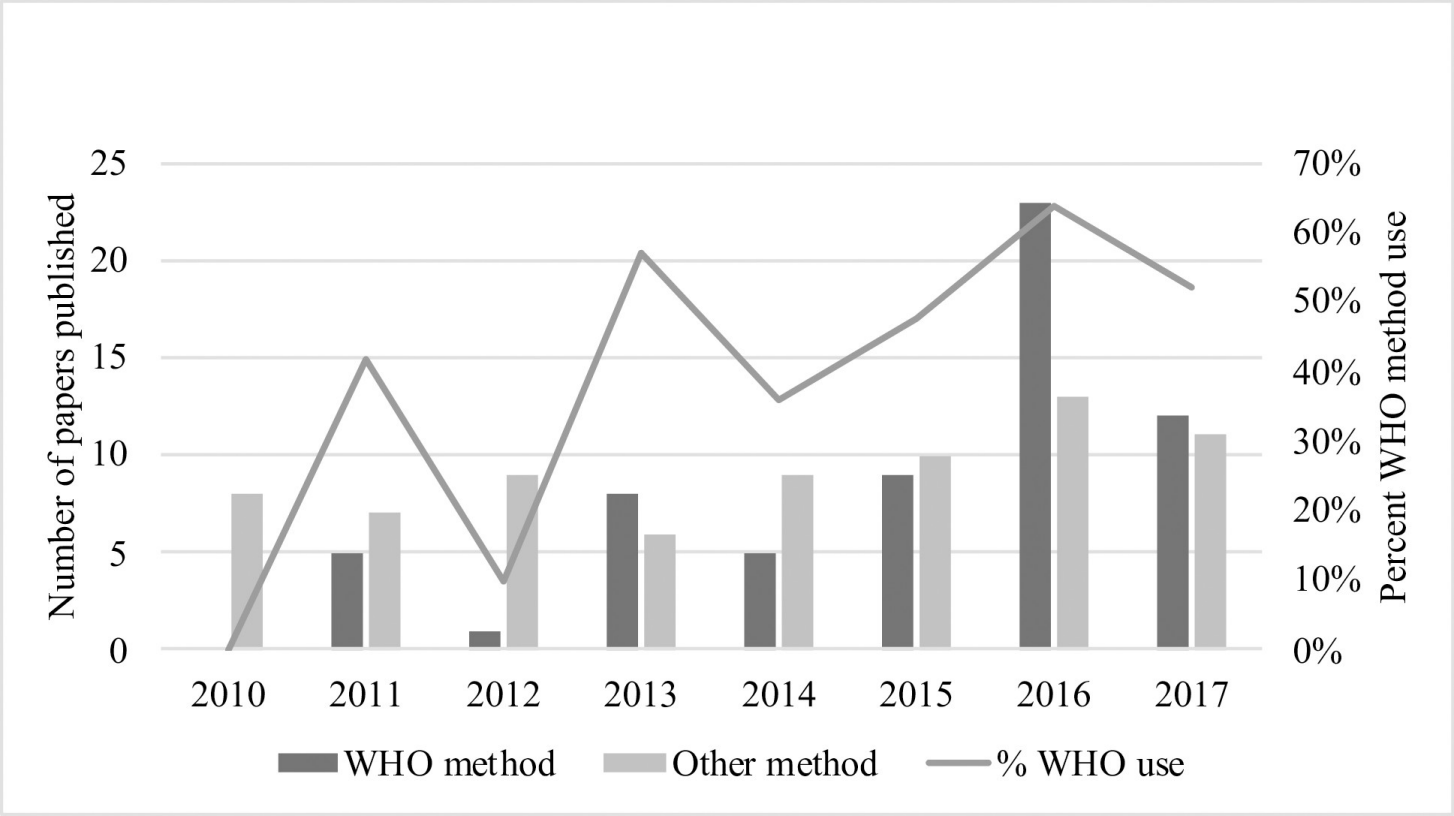
Trends in use of WHO near-miss approach or other methods, 2010–2017.

Overall, the WHO near-miss approach was used in 38.2% (n = 68) studies included in our review, and utilization of this approach has increased over time, with 52.2% of papers published in 2017 using this approach (Fig 3). Importantly, there has been variable uptake of the WHO near-miss approach between world regions. Between 2010 and 2017, the WHO near-miss approach was used in 77.1% (n = 27) of studies evaluating MNM/SMM in Asian countries, 70.0% (n = 21) in African countries, 62.5% (n = 5) in Oceanic countries, 56.5% (n = 13) in South American countries, 14.7% (n = 5) in North American countries, and 6.7% (n = 1) in European countries. Of the five studies that used this approach in North American countries, only one was from the United States, and the remaining four were counted as North American because they evaluated countries in Latin America which spans the continents of both North and South America. Some studies evaluated populations in multiple continents and thus were counted twice in these calculations. Of the 68 papers included in our review that used the WHO near-miss approach, 22.1% (n = 15) used a modified version most of which were due to limitations in their clinical environment; however, two of these used a modified version to broaden the WHO near-miss approach.

### Hospital admissions

Less than half (46.1%, n = 82) of included studies explicitly stated what admissions were included in their data (i.e., antepartum admissions, delivery admissions, postpartum admissions, or a combination of the three). Of the 82 papers that reported on this, nearly half (48.8%, n = 40) evaluated all antepartum, delivery, and postpartum admissions, 39.0% (n = 32) studies evaluated the delivery hospitalization only, 4.9% (n = 4) studies evaluated antepartum and delivery hospitalizations, 2.4% (n = 2) evaluated delivery and postpartum admissions, and 2.4% (n = 2) evaluated only postpartum admissions in their studies.

### Facility type

Of the 178 included papers, 58.4% (n = 104) gathered data at a population level or from multiple centres, one did not specify if they used data from a single or multiple centres), and the remaining portion evaluated a population from a single centre only, of which 31.5% (n = 56) were a tertiary/academic facility, 2.8% (n = 5) a community hospital, and 6.7% (n = 12) from one site without further detail provided.

### Indicator selection method/quality assessment

With regards to the reasons for inclusion of specific indicators, 18.0% (n = 32) of included papers did not provide information on how indicators were chosen, 10.1% (n = 18) described the process for developing a unique set of indicators for their study, 26.4% (n = 47) used indicators developed/identified by previous authors, and 45.5% (n = 81) used indicators recommended by the WHO or CDC, or modifications of these indicators based on limitations in their clinical environment. The processes used by authors in developing a unique set of indicators for their studies varied, with some authors providing details on literature searches performed to examine what indicators have been used in the past, and others discussing conferences with physicians working in maternal healthcare to determine a list of important and relevant indicators for their study.

### Comparison of common surveillance methods

Applying the CDC, CPSS, MMOI, Mantel et al. and Waterstone et al. criteria to the 2016 US National Inpatient Sample (NIS) yielded statistically significant differences in rate of MNM/SMM [8, 9, 12–17]. Using the CDC criteria, the rate was 5.07% (95% CI = 5.02, 5.11), using Waterstone et al. the rate was 5.08% (95% CI = 5.03, 5.12), using Mantel et al. the rate was 6.44% (95% CI = 6.38, 6.49), using the MMOI the rate was 7.32% (95% CI = 7.27, 7.38), and using the CPSS criteria the rate was 7.85% (95% CI = 7.79, 7.91).

## Discussion

Our review sought to assess the terms, reporting systems, and indicators currently being used to describe and characterize events/conditions in the maternal population that are considered significant enough to impact maternal short- and long-term outcomes and thought to be reflective of MNM/SMM. We found substantial overlapping themes in the 198 indicators assessed in studies included in our review, which is likely a reflection of the fact that current monitoring approaches assess different aspects of a patient’s condition when monitoring for a related clinical concept. For example, some included studies assessed haemorrhage, others assessed specific types of haemorrhage (e.g. antepartum or postpartum haemorrhage), others linked a haemorrhage event to a specific type of management (e.g. haemorrhage requiring transfusion, hysterectomy, or other interventions), others assessed these management interventions alone, while still others assessed different sequelae of haemorrhage (e.g. hypovolemic shock or anemia). The fact that there are no standard and specific events/conditions that are currently monitored worldwide limits our ability to truly elucidate trends in these conditions.

We also found variation in the way indicators are worded/coded in different studies; for example, some studies assessed amniotic fluid embolisms using the phrase “amniotic fluid embolism” while others used the phrase “obstetric embolism” to describe an amniotic fluid embolism, and others used the phrase “obstetric embolism” to refer to any embolic event, including but not limited to amniotic fluid embolisms. This is problematic and could lead to false assumptions being made regarding the patterns in events that are entirely different entities yet are being referred to using the same term/phrase. In addition to the fact that further work needs to be done in determining what events/conditions should be used as indicators for MNM/SMM, it is imperative efforts are focused on standardizing the definitions of individual indicators.

We found that the number of studies using the WHO near-miss approach has been increasing since the recommendations were released. However, our review has identified a number of issues with this method of surveillance. Use of the WHO near-miss approach has largely been in low to middle income countries only, with very few studies using this approach in North America or Europe. One possible reason for this pattern in use is that the WHO near-miss approach does not use ICD codes for surveilance, and ICD codes are commonly used in research and routine public health surveillance in developed countries. This difficulty in application of the WHO near-miss approach in research in developed countries is readily apparent in our own study as well, given that we could not use the WHO near-miss approach in our comparison of studies using the NIS data. Souza et al. (2012) validated the WHO near-miss criteria in a large 1-year prospective surveillance study in 27 referral maternity hospitals in Brazil; they found that the WHO near-miss criteria were highly associated with maternal death and accurately identified cases of women who nearly died from a severe complication/event [18]. They also describe a novel method of predicting the unique probability of a maternal death in each individual case of MNM, the maternal severity index [18]. In addition, they discuss the development of a maternal severity score, which could provide valuable information on the level of complexity in a patient population at a specific healthcare facility [18]. They state that this has the potential to influence practices and funding/resources at specific facilities. Importantly, they note that these additional methods of classification were developed using the WHO near-miss criteria in a developing country population thus the utility in developed countries is unknown [18]. It is also important to highlight the fact that, of the 68 papers that used the WHO near-miss approach, over one in five used a modified version. The majority of these were due to lack of resources necessary to monitor MNM/SMM using the WHO near-miss approach (e.g. for a particular laboratory value); however, a few of these studies actually broadened the WHO near-miss approach to ensure they were capturing all cases of significance. One of the expected results of the WHO near-miss approach was that it would provide standardized data from a variety of geographical areas, allowing reliable and comparable results to be obtained [7]. Given the variable uptake and modification systems that have been developed, further work is required to achieve this goal.

There are many benefits to the method of using ICD codes in developed countries; this information is routinely collected which provides timely, standardized, and cost effective access to large amounts of data on the maternal population. These factors make this type of surveillance particuarly simple for large healthcare systems to undertake as there is very little work required by clinicians. However, there is no routine collection of demographic details and information related to social determinants of health which would provide a more complete picture in understanding these events and observed trends in incidence.

Many papers that did not use criteria developed by professional organizations did not provide information on the methods used for determining which indicators they selected for their study. This is problematic, as there is no way to identify or justify why these chosen indicators are representative of MNM/SMM and if other authors then choose to build on this research using the same indicators, the rate of use of unstandardized indicators will increase. Mantel et al., Waterstone et al., and Callaghan et al. were the three most commonly cited authors in the papers that used MNM/SMM surveillance methods described by other authors [4, 13, 14]. While these results seem independent of the uptake of the WHO and CDC methods, both Mantel et al. and Waterstone et al. were used in the development of the WHO near-miss approach, and Callaghan et al. was used by the CDC to develop their list of indicators. These authors’ methods are still being used currently despite the development of standardized organizational approaches based on their work. Further work needs to be done to encourge the use of these methods for consistency and explore reasons why this is not being done at present.

Notably, it was unclear what admissions were being monitored in over half of the studies included in our review. Only a quarter of included studies stated that they included data on postpartum admissions. Given that potential causes of MNM/SMM may only be apparent in postpartum readmissions, such as delayed postpartum haemorrhage or sepsis, it is crucial for studies to include this population. General reporting practices need to be improved so that readers are able to clearly see which admissions are being evaluated in all research on MNM/SMM.

When applying five common methods of MNM/SMM surveillance to the same dataset, we calculated rates of MNM/SMM ranging from 5.07% with the CDC criteria to 7.85% with the CPSS criteria; all of the observed differences in rates were statistically significant, except for the difference between the CDC rate at 5.07% and the Waterstone et al. rate at 5.08%. Given that we analyzed the same dataset using these five different approaches, our finding is a reflection only of the variation in indicators of MNM/SMM. While this is likely not the only factor contributing to varying rates in the literature, it is an issue that can be modified and corrected within the academic and clinical groups studying MNM/SMM and should be a priority.

This study had both strengths and limitations. Our search strategy was broad, with no restrictions on language, date, or location to increase the comprehensiveness of our review. We focused on studies that evaluated MNM/SMM using a composite indicator rather than a single individual condition or event, and extracted data using the language used in each included paper. This provides a clear picture of both minor and major differences in what studies are currently using as indicators for MNM/SMM and may provide an explanation as to why estimates are highly variable. We applied the most common definitions of MNM/SMM to the same data source to quantify the impact that different definitions have on the incidence of MNM/SMM. To our knowledge, ours is the first systematic review on global patterns in indicators used for surveillance of MNM/SMM. While our study did not collect data on the individual studies’ definitions of conditions monitored, we observed substantial variability in this area (e.g. many studies had different cut offs for management or laboratory criteria). Further work is required in this area to collect data on this speficially and to standardize the definitions of each individual indicator of MNM/SMM. While no language restrictions were placed on the search, we were unable to find translations for seven papers which met our inclusion criteria. Given the number of papers included in our review, it is unlikely that this exclusion would have resulted in a substantial impact on our data. We did not find an appropriate tool for assessing the overall quality of included studies; which limits our ability to make specific recommendations on which indicators should be used in monitoring practices. However, the ultimate goal of our review was to illustrate trends in practices currently in use and not to recommend a specific approach, thus the fact that we were unable to perform an adequate quality assessment on included studies does not detract from the overall importance of our findings.

## Conclusion

In conclusion, we found substantial variation in monitoring practices and the language used to describe MNM/SMM, preventing us from being able to conduct international comparisons on MNM/SMM at present. Given the differences in methods employed in developed versus developing countries, it is unclear whether global standardization of MNM/SMM surveillance is feasible at this time. However, there is a clear need to standardize approaches that are used in both of these populations. This would allow better comparisons to be made globally between different geographical areas that have similar patient populations and resources, which could elucidate areas for practice/policy change that would lead to improved care delivery for women in both developed and developing countries.

- Oral presentation at the 75^th^ Annual Clinical Scientific Conference of the Society of Obstetricians and Gynaecologists of Canada–Halifax, NS, Canada, June 13, 2019
- Poster presentation at the 75^th^ Annual Clinical Scientific Conference of the Society of Obstetricians and Gynaecologists of Canada, Medical Student Program–Halifax, NS, Canada, June 10, 2019
- Poster presentation at the John Jarrell Research Day–Calgary, AB, Canada, May 10, 2019
- Poster presentation of methods at the 74^th^ Annual Clinical Scientific Conference of the Society of Obstetricians and Gynaecologists of Canada–Medical Student Program, Victoria, BC, Canada, June 25, 2018

## Supporting information

S1 Appendix Medline search strategy. (DOCX)

S2 Appendix Information contained in data collection tool. (DOCX)

S3 Appendix Maternal near miss/severe maternal morbidity MNM/SMM and associated International Classification of Diseases, Tenth Revision, Clinical Modification ICD-10-CM Codes. (DOCX)

S4 Appendix Study characteristics. (DOCX)
