# Supplementary material for: Monitoring maternal near miss/severe maternal morbidity: A systematic review of global practices
Source: PLoS One. 2020 May 29;15(5):e0233697. doi: 10.1371/journal.pone.0233697 (PMC7259583; doi:10.1371/journal.pone.0233697)
Supplement: S1 Appendix — (DOCX) [file pone.0233697.s001.docx]

MEDLINE Search Strategy

1. Maternal adj2 morbidit* or outcome* or complication*...mp.

2. Severe adj2 maternal adj2 morbidit* or outcome* or complication*....mp.

3. Severe adj2 acute adj2 maternal adj2 morbidit* or outcome* or complication*....mp.

4. Maternal near miss*.mp.

5. Or/1-4

6. Incidence/

7. Public health surveillance/

8. Population surveillance/

9. Prevalence/

10. Incidence.tw,kf.

11. Prevalence.tw,kf.

12. Monitor*.mp.

13. Indicat*.mp.

14. Surveil*.mp.

15. Measur*.mp.

16. Or/6-15

17. 5 and 16
