# Supplementary material for: Monitoring maternal near miss/severe maternal morbidity: A systematic review of global practices
Source: PLoS One. 2020 May 29;15(5):e0233697. doi: 10.1371/journal.pone.0233697 (PMC7259583; doi:10.1371/journal.pone.0233697)
Supplement: S2 Appendix — (DOCX) [file pone.0233697.s002.docx]

| **General information** |
| --- |
| Reviewer |
| Reference ID |
| Authors |
| Year |
| Journal |
| Contact |
| Phrase used |
| Open text area for other phrase used |
| Location |
| Setting   - I.e. single center and level of center or multiple centers |
| Admissions   - I.e. delivery, antepartum, and/or postpartum hospitalizations |
| Admission restrictions   - E.g. gestational age cut off for antepartum admissions, length of time after delivery during which postpartum admissions would be included, etc. |
| Year(s) of data collection |
| Data source |
| Open text area for data source information |
| Design |
| Translation status |
| Open text area for miscellaneous notes |
| Quality |
| Notes on quality |
| Notes on indicators |
| **Surveillance approaches (used for quick data collection)** |
| WHO near-miss approach |
| WHO – modified version |
| CDC |
| CDC – modified version |
| CPSS |
| CPSS – Modified version |
| FLASOG |
| MMOI/Roberts et al. |
| PNG (Papua New Guinea) modified version of WHO |
| Baskett et al. |
| Cairns Base Hospital criteria |
| Callaghan et al. |
| Filippi et al. |
| Geller et al. |
| Haydom/Nelissen modified version of WHO |
| Joseph et al. |
| Main et al. |
| Mantel et al. |
| Prual et al. |
| Roberts et al. |
| Say et al. (WHO criteria published in 2009) |
| Sahel et al. |
| SOFA Score |
| Sousa et al. |
| Souza et al. |
| Waterstone et al. |
| Open text area for other approach if not listed above |
| **Indicators (used for quick data collection)** |
| Abdominal wall infection/peritonitis |
| Abnormal vitals/labs |
| Abortion complications |
| Abruption of placenta |
| Abruption + coagagulation defect |
| Acidosis or pH <7.1 |
| Acute abdomen |
| Acute tubular necrosis |
| Atrial fibrillation or fluttter |
| Amniotic fluid embolism |
| Acute fatty liver of pregnancy |
| Abnormally invasive placenta |
| Abnormally invasive placenta + haemorrhage |
| Anaemia |
| Anesthesia complications |
| Aneurysm |
| Aneurysm - pulmonary artery |
| Antibiotic use |
| Anticonvulsant use |
| Aortic dissection or aortic aneurysm rupture |
| Antepartum haemorrhage |
| Antepartum haemorrhage + coagulation defect |
| Antepartum haemorrhage requiring transfusion |
| Antepartum haemorrhage requiring intervention |
| Appendicitis |
| Acute respiratory distress syndrome or adult respiratory distress syndrome |
| Acute renal failure or acute kidney injury |
| Asthma, severe |
| Azotemia |
| Bladder/urethra repair |
| Bradypnea |
| Budd-Chiari syndrome |
| Cardiac arrest |
| Cardiac disease, complications, events, or procedures – general/unspecified |
| Cardiac disease - stage 4 |
| Cardiac operation |
| Cardiac tamponade |
| Cardio monitoring |
| Cardiomyopathy, peripartum |
| Cardiomyopathy, unspecified |
| Cerebrovascular disorders, including stroke, subarachnoid haemorrhage, intracranial haemorrhage, unspecified haemorrhage, arterial occlusion, or cerebral venous thrombosis |
| Cerebral disorders or complications – general/unspecified |
| Cerebral edema |
| Coagulopathy |
| Collapse, unexplained |
| Coma or prolonged unconsciousness or loss of consciousness |
| Complications of medical or surgical procedure – general/unspecified |
| Conversion of cardiac rhythm |
| Constrictive pericarditis |
| Cor pulmonale |
| Cardiopulmonary resuscitation |
| Creatinine, elevated |
| Caesarean section or emergency caesarean section |
| Cyanosis |
| Dilation and curettage or vacuum aspiration |
| Dehiscence of wound or repair of dehiscence of wound |
| Delirium |
| Diabetes |
| Dialysis or dialysis for acute renal failure or acute kidney injury |
| Disseminated intravascular coagulation |
| Diabetic ketoacidosis or ketoacidosis |
| Deep venous thrombosis |
| Dystocia or obstructed labour |
| Dystocia + complication (including uterine rupture) |
| Dystocia requiring intervention |
| Eclampsia |
| Eclampsia, imminent |
| Eclampsia + jaundice |
| Ectopic pregnancy |
| Ectopic pregnancy + haemorrhage |
| Ectopic pregnancy, ruptured, requiring intervention |
| Embolization, intervention |
| Embolism, including air, thrombus, septic, or obstetric |
| Endocarditis |
| Failure to clot |
| Fluid overload with oxytocin use |
| Gasping |
| Heart failure |
| Haemolysis, elevated liver enzymes, low platelets (HELLP) syndrome |
| Haemolysis, elevated liver enzymes, low platelets (HELLP) syndrome + liver hematoma |
| Haemolysis, elevated liver enzymes, low platelets (HELLP) syndrome + liver rupture |
| Haemorrhage requiring medication or massage |
| Haemorrhage requiring transfusion |
| Haemorrhage requiring hysterectomy or intervention |
| Haemorrhage |
| Haemorrhage + coagulation defect |
| Haematologic disease – general/unspecified |
| Hemopericardium |
| Hepatic disorders – general/unspecified |
| Hepatitis |
| History of pelvic surgery or uterine scar |
| Human immunodeficiency virus |
| Hospitalization greater than 1 week |
| Hospitalization, prolonged |
| Hypertensive crisis |
| Hypertension requiring hospitalization or intervention |
| Hypertension, severe |
| Hyperbilirubinemia |
| Hypertensive disease – pre-existing |
| Hypertensive disease + renal disease – pre-existing |
| Hypertension, gestational |
| Hypertensive disorders of pregnancy |
| Hypertensive disorders – other |
| Hypertension requiring intravenous medication |
| Hypoperfusion or lactate >5 mmol/l |
| Hypoxemia or low oxygen saturation or low PaO2/FiO2 |
| Hysterectomy |
| Hydramnios |
| Intensive care unit admission (or equivalent) |
| Incisional hematoma evacuation or other hematoma evacuation |
| Infection |
| Infection (uterine) requiring hysterectomy or intervention |
| Internal injuries of thorax, abdomen, pelvis |
| Interventional radiology |
| Intestinal obstruction |
| Intracranial injuries |
| Intrapartum haemorrhage |
| Intrapartum haemorrhage + transfusion |
| Intrapartum haemorrhage + hysterectomy or intervention |
| Intrapartum haemorrhage + coagulation defect |
| Intubation |
| Invasive hemodynamic/cardic monitoring |
| Intrauterine fetal demise |
| Jaundice |
| Labour induction or augmentation |
| Laparotomy or surgery or return to operating room/theatre |
| Liver disease, acute |
| Liver dysfunction requiring intensive care unit admission |
| Liver failure |
| Liver hematoma |
| Liver rupture |
| Loss of consciousness |
| Loss of vision – temporary or permanent |
| Malaria |
| Manual removal of placenta |
| Metabolic disorders – general/unspecified |
| Myocardial infarction |
| Molar pregnancy or gestational trophoblastic disease |
| Multisystem failure |
| Multiple medical interventions |
| Neurologic complications/diseases – general/unspecified |
| Non-responsive to fluids, renal clinical criteria |
| Oliguria or oliguria non-responsive to fluids |
| Oxygen use |
| Organ system failure – not specified |
| Other condition or other condition requiring intervention – general/unspecified |
| Paralysis, total or unspecified |
| Perineal trauma – grade 3-4 |
| Pneumonia, aspiration |
| Postpartum haemorrhage |
| Postpartum haemorrhage + coagulation defect |
| Postpartum haemorrhage requiring transfusion |
| Postpartum haemorrhage requiring hysterectomy or intervention |
| Pre-eclampsia |
| Pre-eclampsia + haemolysis, elevated liver enzymes, low platelets (HELLP) syndrome |
| Pre-eclampsia + jaundice |
| Pre-eclampsia+ jaundice + high international normalized ratio (INR) |
| Previa (placenta) |
| Previa (placenta) + haemorrhage or previa (placenta) + haemorrhage + intervention |
| Psychosis/psychiatric morbidiy |
| Pulmonary edema |
| Pulmonary embolism |
| Pulmonary insufficiency after trauma and surgery |
| Pyelonephritis |
| Readmission to hospital |
| Renal disorders/complications – general or unspecified |
| Resuscitation |
| Respiratory arrest |
| Respiratory diseases, complications, disorders – general/unspecified |
| Respiratory distress – clinical criteria |
| Respiratory failure or collapse |
| Respiratory insufficiency |
| Respiratory support |
| Retained placenta |
| Sepsis or septicaemia |
| Sepsis + pulmonary edema or acute/adult respiratory distress syndrome |
| Sepsis requiring intensive care unit admission or hospitalization |
| Sepsis requiring hysterectomy |
| Seizure or uncontrollable fits |
| Shock – general or unspecified |
| Shock – cardiogenic |
| Shock – anaphylactic/anaphylaxis |
| Shock – obstetric |
| Shock – no-responsive to treatment |
| Shock – septic |
| Shock – hypovolemic or haemorrhagic |
| Shock – lung |
| Sickle cell disease with crisis |
| Systemic inflammatory response syndrome |
| Status asthmaticus |
| Status epilepticus |
| Tachypnea |
| Temporary tracheostomy |
| Thrombocytopenia |
| Thrombocytopenia requiring platelet transfusion |
| Thrombotic thrombocytopenic purpura or idiopathic thrombocytopenic purpura or immune thrombocytopenic purpura |
| Thyroid crisis |
| Thyroiditis |
| Thyrotoxicosis |
| Transfer |
| Transfusion |
| Uremia or elevated urea |
| Uterine inversion |
| Uterine rupture |
| Uterine rupture, impending |
| Vasoactive drug use, continuous |
| Ventilation |
| Ventricular fibrillation or flutter |
| Volume expansion with fluids or medications |
| Open text area for other indicators if not listed above |
