# Supplementary material for: Monitoring maternal near miss/severe maternal morbidity: A systematic review of global practices
Source: PLoS One. 2020 May 29;15(5):e0233697. doi: 10.1371/journal.pone.0233697 (PMC7259583; doi:10.1371/journal.pone.0233697)
Supplement: S3 Appendix — (DOCX) [file pone.0233697.s003.docx]

| **Severe Maternal Morbidity Criteria** | **ICD-10-CM diagnosis and procedure codes used for this study** |
| --- | --- |
| **A. Centre for Diseases Control and Prevention CDC.** | |
| 1. Acute myocardial infarction  Aneurysm | I21.01; I21.02; I21.09; I21.11; I21.19; I21.21; I21.29; I21.3; I21.4; I21.9; I21.a1; I21.a9; I22.0; I22.1; I22.2; I22.8; I22.9  I71.00; I71.01; I71.02; I71.03; I71.1; I71.2; I71.3; I71.4; I71.5; 171.6; I71.8; I71.9; I79.0 |
| 2. Acute renal failure | N17.0; N17.1; N17.2; N17.8; N17.9; O90.4  N17 code was used instead of its sub-codes as sub-codes were not used in the dataset. |
| 3. Adult respiratory distress syndrome | J80; J95.1; J95.2; J95.3; J95.821; J95.822; J96.00; J96.01; J96.02 |
| 4. Amniotic fluid embolism | O88.11; O88.12; O88.13 |
| 5. Cardiac arrest/ventricular fibrillation  Conversion of cardiac rhythm | I46.2; I46.8; I46.9; I49.01; I49.02  5A2204Z; 5A12012 |
| 6. Disseminated intravascular coagulation | D65; D68.8; D68.9; O72.3 |
| 7. Eclampsia | O15.00; O15.02; O15.03; O15.1; O15.2; O15.9; O14.22; O14.23 |
| 8. Heart failure/arrest during surgery or procedure | I97.120; I97.121; I97.130; 197.131 |
| 9. Puerperal cerebrovascular disorders | I60.0; I60.1; I60.2; I60.3; I60.4; I60.5; I60.6; I60.7; I60.8; I60.9; I61.0; I61.1; I61.2; I61.3; I61.4; I61.5; I61.6; I61.7; I61.8; I62.0; I62.1; I62.9; I63.0; I63.1; I63.2; I63.3; I63.4; I63.5; I63.6; I63.7; I63.8; I63.9; I65.0; I65.1; I65.2; I65.8; I65.9; I66.0; I66.1; I66.2; I66.3; I66.8; I66.9; I67.1; I67.2; I67.3; I67.4; I67.5; I67.6; I67.7; I67.8; I67.9; I68.0; I68.8; I68.9; O22.51; O22.52; O22.53; I97.810; I97.811; I97.820; I97.821; O873 |
| 10. Pulmonary edema | J81.0; I50.1; I50.20; I50.21; I50.23; I50.30; I50.31; I50.33; I50.40; I50.41; I50.43; I50.9 |
| 11. Severe anesthesia complications | O74.0; I74.1; I74.2; J74.3; O89.01; O89.09; O89.1; O89.2; O85 |
| 12. Sepsis | A40.0; A40.1; A40.3; A40.8; A40.9; A41.0; A41.1; A141.2; A141.3; A141.4; A141.5; A141.51; A141.52; A141.53; A141.59; A141.81; A141.89; A141.9; A32.7 |
| 13. Shock | O75.1; R57.0; R57.1; R57.8; R57.9; R65.21; T78.2; T88.22; T88.6; T81.10; T81.11; T81.19 |
| 14. Sickle cell disease with crisis | D57.00; D57.01; D57.02; D57.211; D57.212; D57.219; D57.411; D57.412; D57.419; D57.811; D57.812; D57.819 |
| 15. Air and thrombotic embolism | O88.011; O88.012; O88.013; O88.014; O88.015; O88.016; O88.017; O88.018; O88.019; O88.02; O88.03; O88.211; O88.212; O88.213; O88.214; O88.215; O88.216; O88.217; O88.218; O88.219; O88.22; O88.23; O88.311; O88.312; O88.313; O88.314; O88.315; O88.316; O88.317; O88.318; O88.319; O88.32; O88.33; O88.81; O88.82; O88.83; I26.0; I26.01; I26.02; I26.09; I26.90; I26.92; I26.99 |
| 16. Blood transfusion | 30233h1; 30233k1; 30233l1; 30233m1; 30233n1; 30233p1; 30233r1; 30233t1; 30240h1; 30240k1; 30240l1; 30240m1; 30240n1; 30240p1; 30240r1; 30240t1; 30240h1; 30243k1; 30243l1; 30243m1; 30243n1; 30243p1; 30243r1; 30243t1; 30233n0; 30233p0; 30240n0; 30240p0; 30243n0; 30243p0 |
| 17. Hysterectomy | UT90ZZ, 0UT94ZZ, 0UT97ZZ, 0UT98ZZ, 0UT9FZZ |
| 18. Temporary tracheostomy  Ventilation | 0B110Z4, 0B110F4, 0B113Z4, 0B113F4, 0B114Z4, 0B114F4  5A1935Z; 5A1945Z; 5A1955Z |
| **SMM rate: 5.07%** | |
| **B. Canadian Perinatal Surveillance System CPSS.** | |
| 1. Pre-existing hypertensive heart disease | O10.1 |
| 2. Pre-existing hypertensive heart and renal disease | O10.3 |
| 3. Eclampsia | O15 |
| 4. Cerebral venous thrombosis in pregnancy | O22.5 |
| 5. Cerebral venous thrombosis in the puerperium | O87.3 |
| 7. Pulmonary, cardiac, and central nervous system complications of anesthesia during pregnancy, labor and delivery and puerperium | - Pulmonary complications of anesthesia during pregnancy O29.0.  - Cardiac complications of anesthesia during pregnancy O29.1.  - Central nervous system complications of anesthesia during pregnancy O29.2.  - Aspiration pneumonitis due to anesthesia during labor and delivery O74.0.  - Other pulmonary complications of anesthesia during labor and delivery O74.1.  - Cardiac complications of anesthesia during labor and delivery O74.2.  - Central nervous system complications of anesthesia during labor and delivery O74.3.  - Pulmonary complications of anesthesia during the puerperium O89.0.  - Cardiac complications of anesthesia during the puerperium O89.1.  - Central nervous system complications of anesthesia during the puerperium O89.2. |
| 8. Placental abruption with coagulation defect | O45.0 |
| 9. Antepartum hemorrhage with coagulation defect | O46.0 |
| 10. Intrapartum hemorrhage with coagulation defect | O67.0 |
| 11. Rupture of uterus before onset of labour | O71.0 |
| 12. Rupture of uterus during labour | O71.1 |
| 13. Obstetric shock | O75.1; R57; T80.5; T88.6 |
| 14. Septicemia during labour | O75.3 |
| 15. Puerperal sepsis | O85 |
| 16. Obstetric embolism | O88 |
| 17. Cardiomyopathy in the puerperium | - Peripartum cardiomyopathy O90.3.  - Cardiomyopathy I42.  - Cardiomyopathy in diseases classified elsewhere I43. |
| 18. Acute renal failure | - Renal failure following labor and delivery O90.4.  - Acute renal failure N17.  - Unspecified kidney failure N19.  - Post-procedural acute. chronic. kidney failure N99.0. |
| 19. Death, obstetric, cause unspecified | O95 |
| 20. Death, obstetric, after 42 days but <1 year after delivery | O96 |
| 21. Death from sequelae of direct obstetric causes | O97 |
| 22. Sudden death, death from unspecified cause | R96; R97; R98; R99 |
| 23. HIV disease | - HIV diseases-symptomatic B20.  - Asymptomatic human immunodeficiency virus HIV. infection status Z21. |
| 24. Cardiac arrest, cardiac failure, myocardial infarction, or pulmonary edema | - Cardiac complication of anesthesia during puerperium O89.1.  - Cardiac complications of anesthesia during labor and delivery O74.2.  - Other complications of obstetric surgery and procedure O75.4.  - ST elevation STEMI. and non-ST elevation NSTEMI. myocardial infraction I21.  - Subsequent ST elevation STEMI. and no-ST NSTEMI. myocardial infraction I22.  - Cardiac arrest I46.  - Heart failure I50.  - Portal vein thrombosis I81. |
| 25. Cerebrovascular diseases: subarachnoid and intracranial hemorrhage, cerebral infarction, stroke | - Nontraumatic subarachnoid hemorrhage I60.  - Nontraumatic intracerebral hemorrhage I61.  - Other and unspecified nontraumatic intracranial hemorrhage I62.  - Cerebral infarction I63. |
| 26. Adult respiratory distress syndrome | J80 |
| 27. Acute abdomen | - Acute appendicitis K35.  - Unspecified appendicitis K37.  - Peritonitis K65.  - Female acute pelvis peritonitis N73.3.  - Female pelvic peritonitis, unspecified N73.5. |
| 28. Hepatic failure | K71; K72 |
| 29. Acute psychosis | - Puerperal psychosis F53.  - Brief psychotic disorder F23. |
| 30. Cerebral edema or coma | - Cerebral edema G93.6.  - Coma R40.2. |
| 31. Disseminated intravascular coagulation | D65 |
| 32. Sickle cell anemia with crisis | D57.0 |
| 33. Status asthmatics | - Mild intermittent asthma with status asthmaticus J45.22.  - Mild persistent asthma with status asthmaticus J45.32.  - Moderate persistent asthma with status asthmaticus J45.42.  - Severe persistent asthma with status asthmaticus J45.52.  - Unspecified asthma with status asthmaticus J45.902. |
| 34. Status epilepticus | - Localization-related focal. partial. idiopathic epilepsy and epileptic syndromes with seizures of localized onset, not intractable, with status epilepticus G40.001.  - Localization-related focal. partial. idiopathic epilepsy and epileptic syndromes with seizures of localized onset, intractable, with status epilepticus G40.011.  - Localization-related focal. partial. symptomatic epilepsy and epileptic syndromes with simple partial seizures, not intractable, with status epilepticus G40.101.  - Localization-related focal. partial. symptomatic epilepsy and epileptic syndromes with simple partial seizures, intractable, with status epilepticus G40.111.  - Localization-related focal. partial. symptomatic epilepsy and epileptic syndromes with complex partial seizures, not intractable, with status epilepticus G40.201.  - Localization-related focal. partial. symptomatic epilepsy and epileptic syndromes with complex partial seizures, intractable, with status epilepticus G40.211.  - Generalized idiopathic epilepsy and epileptic syndromes, not intractable, with status epilepticus G40.301.  - Generalized idiopathic epilepsy and epileptic syndromes, intractable, with status epilepticus G40.311.  - Absence epileptic syndrome, not intractable, with status epilepticus G40.a01.  - Absence epileptic syndrome, intractable, with status epilepticus G40.a11.  - Juvenile myoclonic epilepsy, not intractable, with status epilepticus G40.b01.  - Juvenile myoclonic epilepsy, intractable, with status epilepticus G40.b11.  - Other generalized epilepsy and epileptic syndromes, not intractable, with status epilepticus G40.401.  - Other generalized epilepsy and epileptic syndromes, intractable, with status epilepticus G40.411.  - Epileptic seizures related to external causes, not intractable, with status epilepticus G40.501.  - Other epilepsy, not intractable, with status epilepticus G40.801.  - Other epilepsy, intractable, with status epilepticus G40.803.  - Lennox-Gastaut syndrome, not intractable, with status epilepticus G40.811.  - Lennox-Gastaut syndrome, intractable, with status epilepticus G40.813.  - Epileptic spasms, not intractable, with status epilepticus G40.821.  - Epileptic spasms, intractable, with status epilepticus G40.823.  - Epilepsy, unspecified, not intractable, with status epilepticus G40.901.  - Epilepsy, unspecified, intractable, with status epilepticus G40.911. |
| 35. Assisted ventilation through endotracheal tube  Assisted ventilation through tracheostomy | 5A0935; 5A0945; 5A0955 |
| 36. Total hysterectomy, open approach uterus and surrounding structure.  37. Subtotal hysterectomy, open approach uterus and surrounding structure. | 0UT90ZZ |
| 38. Caesarean hysterectomy | - Caesarean delivery 10D00Z0; 10D00Z1; 10D00Z2.  - Hysterectomy 0UT90ZZ.  Caesarean hysterectomy = caesarean delivery + Hysterectomy |
| 39. Blood transfusion whole blood or red cell transfusion. | 30233H1; 30233N1; 30240H1; 30240N1; 30243N1; 30233N0; 30240N0; 30243N0 |
| 40. Dialysis | - Urinary dialysis single 5A1D00Z.  - Urinary dialysis multiple 5A1D60Z. |
| 41. Evacuation of incisional hematoma | - Caesarean delivery 10D00Z0; 10D00Z1; 10D00Z2.  - Hysterectomy 0UT90ZZ.  - Drainage of uterus, vagina, and vulva 0U990ZZ; 0U9900Z; 0U9G0ZZ; 0U9G00Z; 0U9M0ZZ; 0U9M00Z.  Caesarean delivery or hysterectomy + drainage |
| 42. Repair of bladder, urethra, or intestine | - Repair of bladder 0TQB.  - repair of bladder neck 0TQC.  - repair of urethra 0TQD.  - Repair of small and large intestine 0DQ8; 0DQ9; 0DQA; 0DQB; 0DQC; 0DQE; 0DQF; 0DQG; 0DQH; 0DQJ; 0DQK; 0DQL; 0DQM; 0DQN; 0DQP. |
| 43. Placenta previa with hemorrhage + blood transfusion | - O44.1+ blood transfusion |
| 44. Intrapartum hemorrhage + blood transfusion as above. | - O67 + blood transfusion |
| 45. Postpartum hemorrhage + blood transfusion as above. | - O72 + blood transfusion |
| 46. Postpartum hemorrhage + hysterectomy as above. | - O72 + hysterectomy |
| 47. Embolization or ligation of pelvic vessels or suturing of uterus e.g., B-Lynch suture. + postpartum hemorrhage as above. | - Embolization or occlusion of internal iliac artery, right, uterine artery 04LE3DT.  - Embolization or occlusion of internal iliac artery, left, uterine artery 04LF3DU.  - Ligation of internal iliac artery, right 04QE3ZZ.  - Ligation of internal iliac artery, left 04QF3ZZ.  - Suturing of uterus: Repair of uterus, open 0UQ90ZZ.  O72 + Embolization or ligation or suturing of uterus |
| **SMM rate: 7.85%** | |
| **C. Maternal Morbidity Outcome Indicator MMOI.** | |
| 1. Acute abdomen | - Acute appendicitis K35.  - Unspecified appendicitis K37.  - Peritonitis K65.  - Female acute pelvis peritonitis N73.3.  - Female pelvic peritonitis, unspecified N73.5. |
| 2. Acute renal failure | - Renal failure following labor and delivery O90.4.  - Acute renal failure N17.  - Unspecified kidney failure N19.  - Post-procedural acute. chronic. kidney failure N99.0. |
| 3. Acute psychosis | - Puerperal psychosis F53.  - Brief psychotic disorder F23. |
| 4. Cardiac arrest, failure, infarction | - Cardiac complication of anesthesia during puerperium O89.1.  - Cardiac complications of anesthesia during labor and delivery O74.2.  - Cardiomyopathy in the puerperium O90.3  - Acute myocardial infraction I21.  - Cardiomyopathy I42.  - Cardiomyopathy in disease classified elsewhere I43.  - Cardiac arrest I46.  - Heart failure I50.  - Portal vein thrombosis I81. |
| 5. Cerebral oedema or Coma | - Cerebral edema g93.6.  - Coma r40.2. |
| 6. Disseminated Intravascular Coagulopathy | D65 |
| 7. Cerebrovascular Accident | - Nontraumatic subarachnoid hemorrhage I60.  - Nontraumatic intracerebral hemorrhage I61.  - Other and unspecified nontraumatic intracranial hemorrhage I62.  - Cerebral infarction I63. |
| 8. Major complications of Anesthesia | - Pulmonary complications of anesthesia during pregnancy O29.0.  - Cardiac complications of anesthesia during pregnancy O29.1.  - Central nervous system complications of anesthesia during pregnancy O29.2.  - Aspiration pneumonitis due to anesthesia during labor and delivery O74.0.  - Cardiac complications of anesthesia during labor and delivery O74.2.  - Central nervous system complications of anesthesia during labor and delivery O74.3.  - Pulmonary complications of anesthesia during the puerperium O89.0.  - Cardiac complications of anesthesia during the puerperium O89.1.  - Central nervous system complications of anesthesia during the puerperium O89.2. |
| 9. Obstetric Embolism | O88 |
| 10. Obstetric Shock | O75.1; R57; T80.5; T88.6 |
| 11. Sickle cell anaemia with crisis | D57.0 |
| 12. Status asthmaticus | - Mild intermittent asthma with status asthmaticus J45.22.  - Mild persistent asthma with status asthmaticus J45.32.  - Moderate persistent asthma with status asthmaticus J45.42.  - Severe persistent asthma with status asthmaticus J45.52.  - Unspecified asthma with status asthmaticus J45.902. |
| 13. Status epilepticus | - Localization-related focal. partial. idiopathic epilepsy and epileptic syndromes with seizures of localized onset, not intractable, with status epilepticus G40.001.  - Localization-related focal. partial. idiopathic epilepsy and epileptic syndromes with seizures of localized onset, intractable, with status epilepticus G40.011.  - Localization-related focal. partial. symptomatic epilepsy and epileptic syndromes with simple partial seizures, not intractable, with status epilepticus G40.101.  - Localization-related focal. partial. symptomatic epilepsy and epileptic syndromes with simple partial seizures, intractable, with status epilepticus G40.111.  - Localization-related focal. partial. symptomatic epilepsy and epileptic syndromes with complex partial seizures, not intractable, with status epilepticus G40.201.  - Localization-related focal. partial. symptomatic epilepsy and epileptic syndromes with complex partial seizures, intractable, with status epilepticus G40.211.  - Generalized idiopathic epilepsy and epileptic syndromes, not intractable, with status epilepticus G40.301.  - Generalized idiopathic epilepsy and epileptic syndromes, intractable, with status epilepticus G40.311.  - Absence epileptic syndrome, not intractable, with status epilepticus G40.a01.  - Absence epileptic syndrome, intractable, with status epilepticus G40.a11.  - Juvenile myoclonic epilepsy, not intractable, with status epilepticus G40.b01.  - Juvenile myoclonic epilepsy, intractable, with status epilepticus G40.b11.  - Other generalized epilepsy and epileptic syndromes, not intractable, with status epilepticus G40.401.  - Other generalized epilepsy and epileptic syndromes, intractable, with status epilepticus G40.411.  - Epileptic seizures related to external causes, not intractable, with status epilepticus G40.501.  - Other epilepsy, not intractable, with status epilepticus G40.801.  - Other epilepsy, intractable, with status epilepticus G40.803.  - Lennox-Gastaut syndrome, not intractable, with status epilepticus G40.811.  - Lennox-Gastaut syndrome, intractable, with status epilepticus G40.813.  - Epileptic spasms, not intractable, with status epilepticus G40.821.  - Epileptic spasms, intractable, with status epilepticus G40.823.  - Epilepsy, unspecified, not intractable, with status epilepticus G40.901.  - Epilepsy, unspecified, intractable, with status epilepticus G40.911. |
| 14. Uterus rupture | O71.0; O71.1 |
| 15. Assisted Ventilation including tracheostomy | 5A0935; 5A0945; 5A0955 |
| 16. Curettage with general anesthetic | - Curettage 10D17ZZ.  - General anesthesia 3E0NZ.  - Caesarean delivery 10D00Z0; 10D00Z1; 10D00Z2.  - Vaginal delivery 10E0XZZ; 10D07Z3; 10D07Z4; 10D07Z5; 10D07Z6; 10D07Z7; 10D07Z8  Curettage + caesarean delivery or vaginal delivery + general anesthesia |
| 17. Dialysis | - Urinary dialysis single 5A1D00Z.  - Urinary dialysis multiple 5A1D60Z. |
| 18. Evacuation of hematoma | - Caesarean delivery 10D00Z0; 10D00Z1; 10D00Z2.  - Hysterectomy 0UT90ZZ.  - Drainage of uterus, vagina, and vulva 0U990ZZ; 0U9900Z; 0U9G0ZZ; 0U9G00Z; 0U9M0ZZ; 0U9M00Z.  Caesarean delivery or hysterectomy + drainage |
| 19 Hysterectomy | - Open 0UT90ZZ.  - Percutaneous 0UT94ZZ.  - Via natural or artificial opening 0UT97ZZ.  - Via natural or artificial opening with percutaneous 0UT98ZZ.  - Via natural or artificial opening with percutaneous endoscopic assistance 0UT9FZZ. |
| 20. Procedures to reduce flow of blood to uterus | 04LE3DT; 04LF3DU |
| 21. Repair of bladder or cystostomy | 0TQB; 0TQCT |
| 22. Repair of intestine | 0DQ8; 0DQ9; 0DQA; 0DQB; 0DQC; 0DQE; 0DQF; 0DQG; 0DQH; 0DQJ; 0DQK; 0DQL; 0DQM; 0DQN; 0DQP |
| 23. Repair of ruptured or inverted uterus | - Uterus repair, open 0UQ90ZZ.  - Inverted uterus O71.2.  - Ruptured uterus O71.0, O71.1.  Uterus repair + uterine rupture or uterine inversion |
| 24. Re-closure of disrupted caesarean section wound | - Disrupted caesarean section wound O90.0.  - Uterus repair 0UQ90ZZ.  Uterus repair + disrupted caesarean section wound |
| 25. Transfusion of blood or coagulation factors | 30233H1; 30233K1; 30233L1; 30233M1; 30233N1; 30233P1; 30233R1; 30233T1; 30240h1; 30240k1; 30240l1; 30240M1; 30240N1; 30240P1; 30240R1; 30240T1; 30240H1; 30243K1; 30243L1; 30243M1; 30243N1; 30243P1; 30243R1; 30243T1; 30233N0; 30233P0; 30240N0; 30240P0; 30243N0; 30243P0 |
| **SMM rate: 7.32%** | |
| **D. Mantel et al., 1998** |  |
| 1. Cardiac dysfunction  1.1 Pulmonary oedema  1.2 Cardiac arrest | - Pulmonary edema J81.  - Hypertensive heart disease with heart failure I11.0.  - Dilated cardiomyopathy I42.0.  - Obstructive hypertrophy cardiomyopathy I42.1.  - Other cardiomyopathies I42.8.  - Cardiomyopathy, unspecified I42.9.  - Cardiomyopathy in diseases classified elsewhere I43.  - Cardiac arrest due to underlying cardiac condition I46.2.  - Cardiac arrest due to other underlying condition I46.8.  - Cardiac arrest, cause unspecified I46.9.  - Left ventricular failure I50.1.  - Congestive health failure I50.2, I50.3, I50.4, I50.9.  - Post-procedural cardiac arrest I97.12.  - Intraoperative cardiac arrest I97.71.  - Cardiac arrest following incomplete spontaneous abortion O03.36.  - Cardiac arrest following complete or unspecified spontaneous abortion O03.86.  - Cardiac arrest following induced. termination of pregnancy O04.87.  - Cardiac arrest following failed attempted termination of pregnancy O07.36.  - Cardiac arrest following an ectopic and molar pregnancy O08.81.  - Other complications of obstetric surgery and procedures O75.4.  - Peripartum cardiomyopathy O90.3  - Cardiogenic shock R57.0. |
| 2. Vascular dysfunction  2.1 Hypovolaemia requiring blood | - Volume depletion E86.  - Hypovolemic shock NOS R57.1.  - Cardio-vascular collapse R57.9.  - Shock during or following labor and delivery O75.1.  - Post-procedural hypovolemic shock T81.1. |
| 3. Immunological dysfunction  3.1 Intensive care admission for sepsis  3.2 Emergency hysterectomy for sepsis | - Infection; Sepsis; Abortion complicated by genital tract infection;  Peritonitis; Salpingitis A02.1; A22.7; A26.7; A32.7; A40.0; A40.1; A40.2; A40.3; A40.8; A40.9; A41.0; A41.1; A41.2; A41.3; A41.4; A41.5; A41.8; A41.9; A42.7; A54.8; B37.7; K35.0; K35.9; K65.0; K65.8; K65.9; M86.9; N70.0; N70.9; N71.0; N73.3; N73.5; O03.0; O03.5; O04.0; O04.5; O07.0; O08.0; O08.2; O08.3; O41.1; O75.3; O85 O86.0; O86.8; O88.3; T80.2. |
| 4. Respiratory dysfunction  4.1 Intubation and ventilation for  more than 60 min  4.2 Oxygen saturation <90% lasting  more than 60 min  4.3 Ratio Pa O2/ Fi O2 ≤3 | - Pulmonary embolism without acute cor pulmonale I26.9.  - Acute respiratory distress syndrome J80.  - Acute respiratory failure J96.0.  - Respiratory failure, unspecified J96.9.  - Embolism following complete or unspecified spontaneous abortion O03.7.  - Embolism following induced. termination of pregnancy O04.7.  - Cardiorespiratory failure R09.2. |
| 5. Renal dysfunction  5.1 Oliguria defined as <400 ml/24h | - Renal failure following ectopic and molar pregnancy O08.4.  - Anuria and oliguria R34.  - Disorders of urea cycle metabolism E72.2.  -Hypertensive chronic kidney disease with stage 5 chronic disease or end stage renal disease I12.0.  -Hypertensive heart and chronic disease without heart failure I13.1.  -Hypertensive heart and chronic kidney disease with heart failure with stage 5 chronic disease or end stage renal disease I13.2.  - Acute kidney failure with tubular necrosis N17.0.  - Acute kidney failure with acute cortical necrosis N 17.1.  - Acute kidney failure with medullary necrosis 17.2.  - Other acute kidney failure N17.8.  - Acute kidney failure, unspecified N17.9.  - Renal failure following ectopic and molar pregnancy O08.4.  - Postpartum acute kidney failure O90.4. |
| 5.2 Acute deterioration of urea to  >15 mmol/l or of creatinine to  >400 mmol/l |  |
| 6. Liver dysfunction  6.1 Jaundice in the presence of pre-eclampsia | - Acute and subacute hepatic failure K72.0.  - Hepatic Failure, unspecified K72.9.  - Liver and biliary tract disorders in pregnancy, childbirth and the puerperium O26.6.  - Viral hepatitis complicating pregnancy, childbirth and the puerperium conditions O98.4. |
| 7. Metabolic dysfunction  7.1 Diabetic keto-acidosis  7.2 Thyroid crisis | - Type 1 diabetes mellitus with ketoacidosis without coma E10.10.  - Type 1 diabetes mellitus with ketoacidosis with coma E10.11.  - Other specified diabetes mellitus with ketoacidosis without coma 13.10.  - Other specified diabetes mellitus with ketoacidosis with coma E13.11.  - Thyrotoxicosis with diffuse goiter E05.0.  - Thyrotoxicosis with toxic single thyroid nodule E05.1.  - Thyrotoxicosis with toxic multinodular goiter E05.2.  - Thyrotoxicosis from ectopic thyroid tissue E05.3.  - Thyrotoxicosis factitia E05.4.  - Other thyrotoxicosis E05.8.  - Thyrotoxicosis, unspecified E05.9.  - Acute thyroiditis E06.0.  - Hypersecretion of calcitonin E07.0.  - Other specified disorders of thyroid E07.8.  - Disorder of thyroid, unspecified E07.9.  - Metabolic disorders following an ectopic and molar pregnancy O08.5. |
| 8. Coagulation dysfunction  8.1 Acute thrombocytopenia  requiring platelet transfusion | - Disseminated intravascular coagulation D65.  - Other coagulation defects D68.  - Other primary thrombocytopenia D69.4.  - Secondary thrombocytopenia D69.5.  - Thrombocytopenia, unspecified D69.6.  - Wiskott-Aldrich syndrome D82.0.  - Premature separation of placenta with coagulation defect O45.0.  - Postpartum coagulation defects O72.3. |
| 9. Cerebral dysfunction  9.1 Coma lasting > 12 h  9.2 Subarachnoid or intracerebral  haemorrhage | - Cerebral edema G93.6.  - Nontraumatic subarachnoid hemorrhage I60.  - Nontraumatic intracerebral hemorrhage I61.  - Cerebral infarction, unspecified Stroke NOS. I63.9.  - Sequelae of nontraumatic intracerebral hemorrhage I69.1.  - Cerebral venous thrombosis in pregnancy O22.5. |
| 10. Intensive care admission  10.1 For any reason | - No data in the database |
| 11. Emergency hysterectomy  11.1 For any reason | - Caesarean delivery 10D00Z0; 10D00Z1; 10D00Z2.  - Hysterectomy 0UT90ZZ.  Caesarean delivery + hysterectomy |
| 12. Anaesthetic accidents  12.1 Severe hypotension associated  with a spinal or epidural anaesthetic  12.2 Failed tracheal intubation  requiring anaesthetic reversal | - Pulmonary complications of anesthesia during pregnancy O29.0.  - Cardiac complications of anesthesia during pregnancy O29.1.  - Central nervous system complications of anesthesia during pregnancy O29.2.  - Toxic reaction to local anesthesia during pregnancy O29.3.  - Other complications of spinal and epidural anesthesia during pregnancy O29.5.  - Other complications of anesthesia during pregnancy O29.8.  - Unspecified complication of anesthesia during pregnancy O29.9.  - Aspiration pneumonitis due to anesthesia during labor and delivery O74.0.  - Other pulmonary complications of anesthesia during labor and delivery O74.1.  - Cardiac complications of anesthesia during labor and delivery O74.2.  - Central nervous system complications of anesthesia during labor and delivery O74.3.  - Toxic reaction to local anesthesia during labor and delivery O74.4.  - Other complications of spinal and epidural anesthesia during labor and delivery O74.6.  - Other complications of anesthesia during labor and delivery O74.8.  - Complication of anesthesia during labor and delivery, unspecified O74.9.  - Pulmonary complications of anesthesia during the puerperium O89.0.  - Cardiac complications of anesthesia during the puerperium O89.1.  - Central nervous system complications of anesthesia during the puerperium O89.2.  - Toxic reaction to local anesthesia during the puerperium O89.3.  - Other complications of spinal and epidural anesthesia during the puerperium O89.5.  - Other complications of anesthesia during the puerperium O89.8.  - Complication of anesthesia during the puerperium, unspecified O89.9.  - Shock due to anesthesia T88.2.  - Malignant hyperthermia due to anesthesia T83.3.  - Other complications of anesthesia T88.5. |
| **SMM rate: 6.44%** | |
| **E. Waterstone et al., 2001** |  |
| 1. Severe pre-eclampsia | - Mild, severe or unspecified pre-eclampsia; Pre-existing hypertensive disorder with superimposed proteinuria O11; O14.0; O14.1; O14.9. |
| 2. Eclampsia | - Eclampsia in pregnancy, labor or delivery O15; O15.0; O15.1; 15.2; O15.9. |
| 3. Hemolysis, elevated liver enzymes, low platelets syndrome | - Hemolysis, elevated liver enzymes, low platelets syndrome O14.2. |
| 4. Severe hemorrhage | - Acute post-hemorrhagic anemia D62.  - Delayed or excessive hemorrhage following incomplete spontaneous abortion O03.1.  - Delayed or excessive hemorrhage following complete or unspecified spontaneous abortion O03.6.  - Delayed or excessive hemorrhage following induced. termination of pregnancy  O04.6.  - Delayed or excessive hemorrhage following failed attempted termination of pregnancy O07.1.  - Delayed or excessive hemorrhage following ectopic and molar pregnancy O08.1.  - Placenta previa with hemorrhage O44.1.  - Premature separation of placenta with coagulation defect O45.0.  - Other premature separation of placenta O45.8.  - Premature separation of placenta, unspecified O45.9.  - Antepartum hemorrhage with coagulation defect 046.0.  - Other antepartum hemorrhage 046.8.  - Antepartum hemorrhage, unspecified 046.9.  - Intrapartum hemorrhage with coagulation defect O67.0.  - Other intrapartum hemorrhage O67.8.  - Intrapartum hemorrhage, unspecified O67.9.  - Labor and delivery complicated by vasa previa O69.4.  - Postpartum hemorrhage O72, or O72.0 - O72.2. |
| 5. Severe sepsis | - Infection; Sepsis; Abortion complicated by genital tract infection;  Peritonitis; Salpingitis A02.1; A22.7; A26.7; A32.7; A40.0; A40.1; A40.2; A40.3; A40.8; A40.9; A41.0; A41.1; A41.2; A41.3; A41.4; A41.5; A41.8; A41.9; A42.7; A54.8; B37.7; K35.0; K35.9; K65.0; K65.8; K65.9; M86.9; N70.0; N70.9; N71.0; N73.3; N73.5; O03.0; O03.5; O04.0; O04.5; O07.0; O08.0; O08.2; O08.3; O41.1; O75.3; O85 O86.0; O86.8; O88.3; T80.2. |
| 6. Uterine rupture | - Rupture of uterus before or during labor O71.0; O71.1.  - Disruption of caesarean delivery wound O90.0. |
| **SMM rate: 5.08%** | |
