# Supplementary material for: Monitoring maternal near miss/severe maternal morbidity: A systematic review of global practices
Source: PLoS One. 2020 May 29;15(5):e0233697. doi: 10.1371/journal.pone.0233697 (PMC7259583; doi:10.1371/journal.pone.0233697)
Supplement: S4 Appendix — (DOCX) [file pone.0233697.s004.docx]

| **Authors** | **Publication Year** | **Location** | **Admission Type** | **Population Restrictions** | **Setting** | **Method of Selecting Indicators** |
| --- | --- | --- | --- | --- | --- | --- |
| Abha et al.[19] | 2016 | India | Did not explicitly state | None stated | Single site, level not specified | WHO |
| Adeoye et al.[20] | 2013 | Nigeria | Antepartum, delivery, and postpartum | Antepartum admissions in third trimester only, postpartum admissions up to 42 days | Tertiary/academic facility or equivalent | Filippi et al. |
| Almerie et al.[21] | 2010 | Syria | Did not explicitly state | Up to 42 days postpartum | Tertiary/academic facility or equivalent | Filippi et al. |
| Amaral et al.[22] | 2011 | Brazil | Did not explicitly state | Up to 42 days postpartum | Multiple sites/population level | Mantel et al., Waterstone et al. |
| Angelini et al.[23] | 2018 | Brazil | Did not explicitly state | None stated | Unclear | WHO |
| Assarag et al.[24] | 2015 | Morocco | Did not explicitly state | Up to 42 days postpartum | Multiple sites/population level | Sahel et al. |
| Bakshi et al.[25] | 2015 | India | Antepartum, delivery, and postpartum | Up to 42 days postpartum | Multiple sites/population level | WHO |
| Bakshi et al.[26] | 2016 | India | Antepartum, delivery, and postpartum | Up to 42 days postpartum | Multiple sites/population level | WHO |
| Bashour et al.[27] | 2015 | Egypt, Lebanon, Palestine, Syria | Did not explicitly state | No restriction on gestational age, up to 1 week postpartum | Multiple sites/population level | WHO |
| Bateman et al.[28] | 2013 | United States | Delivery | Up to 30 days postpartum, in delivery hospitalization | Multiple sites/population level | Author defined |
| Belanoff et al.[29] | 2016 | United States | Delivery | None stated | Multiple sites/population level | CDC |
| Bolnga et al.[30] | 2017 | Papua New Guinea | Did not explicitly state | None stated | Community hospital or equivalent | WHO, WHO-mod PNG. |
| Booker et al.[31] | 2018 | United States | Delivery | None stated | Multiple sites/population level | CDC |
| Bouvier-Colle et al.[32] | 2012 | 25 countries in the EU and Norway | Did not explicitly state | None stated | Multiple sites/population level | Europeristat |
| Brace et al.[33] | 2004 | Scotland | Did not explicitly state | None stated | Multiple sites/population level | Mantel et al. |
| Brandt et al.[34] | 2014 | United States | Did not explicitly state | None stated | Tertiary/academic facility or equivalent | Callaghan et al. |
| Brown et al.[35] | 2011 | United States | Delivery | None stated | Tertiary/academic facility or equivalent | WHO |
| Callaghan et al.[36] | 2008 | United States | Delivery | None stated | Multiple sites/population level | Callaghan et al., Kuklina et al. |
| Callaghan et al.[4] | 2012 | United States | Delivery and postpartum | None stated | Multiple sites/population level | Geller et al., Wen et al. |
| Calvo-Aguilar et al.[37] | 2010 | Mexico | Did not explicitly state | None stated | Single site, level not specified | FLASOG |
| Camargo et al.[38] | 2011 | Brazil | Did not explicitly state | None stated | Tertiary/academic facility or equivalent | Author defined |
| Cecatti et al.[39] | 2011 | Brazil | Antepartum, delivery, and postpartum | None stated | Tertiary/academic facility or equivalent | WHO-mod, SOFA score |
| Cecatti et al.[40] | 2015 | Brazil | Did not explicitly state | None stated | Multiple sites/population level | Souza et al. |
| Cham et al.[41] | 2009 | Gambia | Did not explicitly state | None stated | Multiple sites/population level | Prual et al. |
| Chantry et al.[42] | 2011 | France | Antepartum, delivery, and postpartum | None stated | Multiple sites/population level | Author defined |
| Chaudhuri et al.[43] | 2018 | India | Antepartum, delivery, and postpartum | Up to 42 days postpartum | Tertiary/academic facility or equivalent | Mantel et al. |
| Chhabra et al.[44] | 2008 | India | Antepartum, delivery, and postpartum | None stated | Tertiary/academic facility or equivalent | Author defined |
| Colmorn et al.[45] | 2015 | Denmark, Finland, Iceland, Norway, Sweden | Did not explicitly state | None stated | Multiple sites/population level | Author defined |
| Creanga et al.[46] | 2014 | United States | Delivery | None stated | Multiple sites/population level | Callaghan et al. |
| Cromi et al.[47] | 2018 | Italy | Did not explicitly state | None stated | Tertiary/academic facility or equivalent | WHO |
| Das et al.[48] | 2014 | India | Postpartum | None stated | Tertiary/academic facility or equivalent | Author defined |
| David et al.[49] | 2014 | Mozambique | Did not explicitly state | None stated | Multiple sites/population level | Author defined |
| De Mucio et al.[50] | 2016 | 12 countries in Latin America | Antepartum, delivery, and postpartum | Up to 42 days postpartum | Multiple sites/population level | WHO |
| De Souza et al.[51] | 2015 | Brazil | Antepartum, delivery, and postpartum | None stated | Tertiary/academic facility or equivalent | Geller et al., Mantel et al., Waterstone et al. |
| Dias et al.[52] | 2014 | Brazil | Did not explicitly state | Greater than 22 weeks gestation or 500 gram birthweight | Multiple sites/population level | WHO |
| El Ghardallou et al.[53] | 2016 | Tunisia | Antepartum, delivery, and postpartum | Up to 42 days postpartum | Tertiary/academic facility or equivalent | WHO |
| Farchi et al.[54] | 2010 | Italy | Did not explicitly state | None stated | Multiple sites/population level | Author defined |
| Frederiksen et al.[55] | 2017 | United States | Delivery | None stated | Multiple sites/population level | Callaghan et al. |
| Friedman et al.[56] | 2016 | United States | Delivery | None stated | Multiple sites/population level | Author defined |
| Furuta et al.[57] | 2014 | England | Did not explicitly state | Greater than 24 weeks gestation, live birth only | Single site, level not specified | Author defined |
| Galindo Mateu et al.[58] | 2010 | Spain | Did not explicitly state | Up to 42 days postpartum | Tertiary/academic facility or equivalent | Baskett et al., Waterstone et al. |
| Galvao et al.[59] | 2014 | Brazil | Antepartum, delivery, and postpartum | No restrictions in gestational age, up to 42 days postpartum | Multiple sites/population level | WHO |
| Gandhi et al.[60] | 2004 | South Africa | Antepartum, delivery, and postpartum | Up to 42 days postpartum | Multiple sites/population level | Mantel et al. |
| Gebrehiwot et al.[61] | 2014 | Ethiopia | Antepartum, delivery, and postpartum | Up to 42 days postpartum | Multiple sites/population level | Author defined |
| Geller et al.[62] | 2002 | United States | Did not explicitly state | None stated | Tertiary/academic facility or equivalent | Author defined |
| Ghazal-Aswad et al.[63] | 2013 | United Arab Emirates | Did not explicitly state | None stated | Multiple sites/population level | Author defined |
| Ghazivakili et al.[64]^.^ | 2016 | Iran | Did not explicitly state | Up to 42 days postpartum | Multiple sites/population level | WHO |
| Gibson et al.[65] | 2017 | United States | Delivery | None stated | Multiple sites/population level | Callaghan et al. |
| Girard et al.[66] | 2001 | France | Did not explicitly state | None stated | Multiple sites/population level | Author defined |
| Goldenberg et al.[67] | 2017 | Republic of Congo, Guatemala, India, Kenya, Pakistan, Zambia | Did not explicitly state | Greater than 20 weeks gestation, up to 42 days postpartum | Multiple sites/population level | WHO-mod Global Network Near-Miss Maternal Mortality System. |
| Haddad et al.[68] | 2011 | Brazil | Did not explicitly state | None stated | Multiple sites/population level | WHO |
| Hassan et al.[69] | 2015 | Palestine | Antepartum, delivery, and postpartum | Up to 42 days postpartum | Single site, level not specified | WHO |
| Herklots et al.[70] | 2017 | Tanzania | Antepartum, delivery, and postpartum | Up to 42 days postpartum | Single site, level not specified | WHO |
| Howell et al.[71] | 2016 | United States | Delivery | None stated | Multiple sites/population level | CDC |
| Howell et al.[72] | 2016 | United States | Delivery | None stated | Multiple sites/population level | CDC |
| Howell et al.[73] | 2014 | United States | Delivery | None stated | Multiple sites/population level | CDC |
| Iwuh et al.[74] | 2018 | South Africa | Did not explicitly state | None stated | Multiple sites/population level | WHO |
| Jabir et al.[75] | 2013 | Iraq | Did not explicitly state | Up to 42 days postpartum | Multiple sites/population level | WHO |
| Jayaratnam et al.[76] | 2016 | Australia | Antepartum, delivery, and postpartum | Up to 42 days postpartum | Tertiary/academic facility or equivalent | WHO, WHO-mod Cairns Base Hospital. |
| Jayaratnam et al.[77] | 2011 | Australia | Antepartum and delivery | None stated | Tertiary/academic facility or equivalent | WHO, WHO-mod Cairns Base Hospital. |
| Joseph et al.[7] | 2010 | Canada | Delivery | None stated | Multiple sites/population level | Author defined |
| Kalisa et al.[78] | 2016 | Rwanda | Antepartum, delivery, and postpartum | Up to 42 days postpartum | Community hospital or equivalent | WHO-mod |
| Kayem et al.[6] | 2011 | United Kingdom | Did not explicitly state | None stated | Multiple sites/population level | Author defined |
| Kiruja et al.[79] | 2017 | Somaliland | Antepartum, delivery, and postpartum | Up to 42 days postpartum | Tertiary/academic facility or equivalent | WHO |
| Knight et al.[80] | 2009 | United Kingdom | Did not explicitly state | None stated | Multiple sites/population level | Author defined |
| Kulkarni et al.[81] | 2016 | India | Did not explicitly state | From conception up to 42 days postpartum | Tertiary/academic facility or equivalent | WHO |
| Lazariu et al.[82] | 2017 | United States | Delivery | None stated | Multiple sites/population level | CDC-mod New York Measure of SMM. |
| Letois et al.[83] | 2016 | France | Antepartum, delivery, and postpartum | Hospitalizations after 24 weeks gestation and up to 42 days postpartum | Multiple sites/population level | Waterstone et al. |
| Linard et al.[84] | 2018 | France | Delivery | None stated | Multiple sites/population level | Author defined |
| Lindquist et al.[85] | 2013 | Australia | Did not explicitly state | None stated | Multiple sites/population level | MMOI |
| Lindquist et al.[86] | 2015 | Australia | Did not explicitly state | None stated | Multiple sites/population level | Author defined |
| Lindquist et al.[87] | 2015 | Australia | Did not explicitly state | None stated | Multiple sites/population level | Author defined |
| Lisonkova et al.[88] | 2017 | United States | Delivery | None stated | Multiple sites/population level | CPSS-mod |
| Lisonkova et al.[89] | 2017 | United States | Delivery | None stated | Multiple sites/population level | CPSS-mod |
| Litorp et al.[90] | 2016 | Tanzania | Antepartum and delivery | 28 weeks gestation and up | Tertiary/academic facility or equivalent | WHO |
| Liu et al.[15] | 2010 | Canada | Did not explicitly state | None stated | Multiple sites/population level | Author defined |
| Liyew et al.[91] | 2017 | Ethiopia | Antepartum, delivery, and postpartum | Up to 42 days postpartum | Multiple sites/population level | WHO |
| Lori et al.[92] | 2012 | Liberia, West Africa | Antepartum, delivery, and postpartum | Up to 42 days postpartum | Community hospital or equivalent | Author defined |
| Lotufo et al.[93] | 2012 | Brazil | Antepartum, delivery, and postpartum | None stated | Tertiary/academic facility or equivalent | WHO |
| Lutomski et al.[94] | 2012 | Ireland and Australia | Delivery | None stated | Multiple sites/population level | Roberts et al. |
| Luz et al.[95] | 2008 | Brazil | Did not explicitly state | None stated | Tertiary/academic facility or equivalent | Mantel et al., Waterstone et al. |
| Lynch et al.[96] | 2008 | England | Did not explicitly state | None stated | Single site, level not specified | Mantel et al. |
| Madeiro et al.[97] | 2015 | Brazil | Antepartum, delivery, and postpartum | Up to 42 days postpartum | Tertiary/academic facility or equivalent | WHO |
| Magalhaes et al.[98] | 2012 | Brazil | Antepartum, delivery, and postpartum | None stated | Multiple sites/population level | Author defined |
| Main et al.[99] | 2016 | United States | Delivery | None stated | Multiple sites/population level | CDC |
| Main et al.[100] | 2017 | United States | Delivery | None stated | Multiple sites/population level | CDC |
| Mantel et al.[13] | 1998 | South Africa | Did not explicitly state | Up to 42 days postpartum | Multiple sites/population level | Author defined |
| Marcellin et al.[101] | 2018 | France | Did not explicitly state | None stated | Tertiary/academic facility or equivalent | Author defined |
| Martin et al.[102] | 2016 | United States | Delivery | None stated | Multiple sites/population level | Callaghan et al. |
| Mawarti et al.[103] | 2017 | Indonesia | Antepartum, delivery, and postpartum | Up to 42 days postpartum | Tertiary/academic facility or equivalent | WHO |
| Mbachu et al.[104] | 2017 | Nigeria | Antepartum and delivery | None stated | Tertiary/academic facility or equivalent | WHO |
| Mekango et al.[105] | 2017 | Ethiopia | Did not explicitly state | None stated | Multiple sites/population level | Filippi et al. |
| Metcalfe et al.[106] | 2018 | United States | Delivery | None stated | Multiple sites/population level | CDC |
| Mohammadi et al.[107] | 2016 | Iran | Did not explicitly state | Up to 42 days postpartum | Multiple sites/population level | WHO-mod |
| Mohammadi et al.[108] | 2017 | Iran | Did not explicitly state | None stated | Multiple sites/population level | WHO-mod |
| Moraes et al.[109] | 2011 | Brazil | Antepartum, delivery, and postpartum | Up to 42 days postpartum | Multiple sites/population level | Mantel et al., Waterstone et al. |
| Muraca et al.[110] | 2017 | Canada | Delivery | None stated | Multiple sites/population level | Author defined |
| Muraca et al.[111] | 2018 | Canada | Delivery | None stated | Multiple sites/population level | Author defined |
| Murphy et al.[112] | 2009 | Ireland | Did not explicitly state | Up to 42 days postpartum | Multiple sites/population level | Mantel et al. |
| Naderi et al.[113] | 2015 | Iran | Delivery and postpartum | Up to 42 days postpartum | Multiple sites/population level | WHO-mod |
| Nair et al.[114] | 2014 | United Kingdom | Did not explicitly state | None stated | Multiple sites/population level | Author defined |
| Nair et al.[115] | 2016 | England | Did not explicitly state | None stated | Multiple sites/population level | Author defined |
| Nakamura-Pereira et al.[116] | 2013 | Brazil | Did not explicitly state | None stated | Multiple sites/population level | WHO |
| Nakimuli et al.[117] | 2015 | Uganda | Did not explicitly state | None stated | Tertiary/academic facility or equivalent | WHO |
| Nakimuli et al.[118] | 2016 | Uganda | Did not explicitly state | None stated | Tertiary/academic facility or equivalent | WHO |
| Nakimuli et al.[119] | 2016 | Uganda | Did not explicitly state | None stated | Tertiary/academic facility or equivalent | WHO |
| Nansubuga et al.[120] | 2016 | Uganda | Did not explicitly state | None stated | Community hospital or equivalent | WHO-mod |
| Nelissen et al.[121] | 2013 | Tanzania | Did not explicitly state | Up to 42 days postpartum | Tertiary/academic facility or equivalent | WHO-mod Haydom/Nelissen. |
| Norhayati et al.[122] | 2016 | Malaysia | Postpartum | At least 22 weeks gestation, up to 42 days postpartum | Tertiary/academic facility or equivalent | WHO |
| Norhayati et al.[123] | 2016 | Malaysia | Did not explicitly state | At least 22 weeks gestation, up to 42 days postpartum | Tertiary/academic facility or equivalent | WHO |
| Norhayati et al.[124] | 2017 | Malaysia | Did not explicitly state | Up to 42 days postpartum | Tertiary/academic facility or equivalent | WHO |
| Norhayati et al.[125] | 2017 | Malaysia | Did not explicitly state | None stated | Tertiary/academic facility or equivalent | WHO |
| O’Malley et al.[126] | 2016 | Ireland | Antepartum, delivery, and postpartum | None stated | Tertiary/academic facility or equivalent | WHO, Scottish SMM criteria |
| Olagbuji et al.[127] | 2012 | Nigeria | Did not explicitly state | None stated | Community hospital or equivalent | Mantel et al. |
| Oliveira et al.[128] | 2013 | Brazil | Antepartum, delivery, and postpartum | None stated | Tertiary/academic facility or equivalent | WHO |
| Ozimek et al.[129] | 2016 | United States | Delivery | None stated | Multiple sites/population level | CDC-mod |
| Pacheco et al.[130] | 2014 | Brazil | Antepartum, delivery, and postpartum | None stated | Tertiary/academic facility or equivalent | WHO |
| Pallasmaa et al.[131] | 2008 | Finland | Delivery | At least 22 weeks gestation, up to 42 days postpartum | Multiple sites/population level | Author defined |
| Pallasmaa et al.[132] | 2015 | Finland | Delivery | At least 22 weeks gestation, up to 42 days postpartum | Multiple sites/population level | Author defined |
| Panday et al.[133] | 2004 | South Africa | Did not explicitly state | None stated | Tertiary/academic facility or equivalent | Mantel et al. |
| Pandey et al.[134] | 2014 | India | Antepartum, delivery, and postpartum | Up to 42 days postpartum | Tertiary/academic facility or equivalent | WHO-mod |
| Parmar et al.[135] | 2016 | India | Did not explicitly state | None stated | Tertiary/academic facility or equivalent | WHO, Mantel et al. |
| Pattinson et al.[136] | 2003 | South Africa | Did not explicitly state | None stated | Multiple sites/population level | Mantel et al. |
| Prual et al.[137] | 1998 | Niger | Antepartum, delivery, and postpartum | At least 28 weeks gestation, up to 42 days postpartum | Multiple sites/population level | WHO 1989 |
| Prual et al.[138] | 2000 | West Africa | Did not explicitly state | None stated | Multiple sites/population level | WHO 1989 |
| Ps et al.[139] | 2013 | India | Did not explicitly state | None stated | Multiple sites/population level | WHO |
| Rabia et al.[140] | 2011 | Pakistan | Antepartum, delivery, and postpartum | Up to 42 days postpartum | Tertiary/academic facility or equivalent | WHO |
| Rana et al.[141] | 2013 | Nepal | Did not explicitly state | None stated | Multiple sites/population level | WHO |
| Rathod et al.[142] | 2016 | India | Antepartum, delivery, and postpartum | None stated | Tertiary/academic facility or equivalent | WHO |
| Reena et al.[143] | 2018 | India | Did not explicitly state | None stated | Single site, level not specified | WHO |
| Reid et al.[144] | 2018 | United States | Delivery | None stated | Multiple sites/population level | CDC |
| Reime et al.[145] | 2012 | Germany | Delivery | None stated | Multiple sites/population level | Author defined |
| Roberts et al.[146] | 2009 | Australia | Delivery | None stated | Multiple sites/population level | MMOI |
| Ronsmans et al.[147] | 2016 | Benin, Burkina Faso, Morocco | Delivery | None stated | Multiple sites/population level | WHO, Filippi et al. |
| Roost et al.[148] | 2009 | Bolivia | Antepartum, delivery, and postpartum | None stated | Multiple sites/population level | Filippi et al. |
| Rosendo et al.[149] | 2015 | Brazil | Did not explicitly state | None stated | Multiple sites/population level | Souza et al. |
| Rulisa et al.[150] | 2015 | Rwanda | Did not explicitly state | None stated | Single site, level not specified | WHO-mod |
| Sahijwani et al.[151] | 2013 | India | Did not explicitly state | Up to 42 days postpartum | Single site, level not specified | Mantel et al. |
| Sangeeta et al.[152] | 2015 | India | Antepartum, delivery, and postpartum | None stated | Tertiary/academic facility or equivalent | WHO |
| Sayinzoga et al.[153] | 2017 | Rwanda | Antepartum, delivery, and postpartum | None stated | Multiple sites/population level | WHO-mod Haydom/Nelissen. |
| Serruya et al.1[154] | 2017 | 12 countries in Latin America and the Caribbean | Did not explicitly state | None stated | Multiple sites/population level | WHO |
| Shields et al.[155] | 2017 | United States | Did not explicitly state | None stated | Multiple sites/population level | CDC |
| Shields et al.[156] | 2016 | United States | Did not explicitly state | None stated | Multiple sites/population level | CDC-mod |
| Shrestha et al.[157] | 2010 | Nepal | Did not explicitly state | Up to 42 days postpartum | Tertiary/academic facility or equivalent | Geller et al. |
| Siddiqui et al.[158] | 2012 | Pakistan | Did not explicitly state | None stated | Tertiary/academic facility or equivalent | Waterstone et al. |
| Sigakis et al.[159] | 2016 | United States | Did not explicitly state | None stated | Multiple sites/population level | Author defined |
| Sikder et al.[160] | 2014 | Bangladesh | Did not explicitly state | None stated | Multiple sites/population level | Author defined |
| Silva et al.[161] | 2016 | Brazil | Antepartum, delivery, and postpartum | None stated | Multiple sites/population level | Sousa et al. |
| Silveira et al.[162] | 2018 | Brazil | Did not explicitly state | None stated | Multiple sites/population level | WHO |
| Soma-Pillay et al.[163] | 2015 | South Africa | Did not explicitly state | None stated | Multiple sites/population level | WHO |
| Sousa et al.[15] | 2008 | Brazil | Antepartum, delivery, and postpartum | None stated | Multiple sites/population level | Author defined |
| Souza et al.[164] | 2013 | 29 countries in Africa, Asia, Latin America, and Middle East | Did not explicitly state | Up to 7 days postpartum | Multiple sites/population level | Author defined |
| Souza et al.[165] | 2010 | Brazil | Did not explicitly state | None stated | Tertiary/academic facility or equivalent | Mantel et al., Waterstone et al. |
| Souza et al.[166] | 2007 | Brazil | Did not explicitly state | None stated | Tertiary/academic facility or equivalent | Author defined |
| Souza et al.[167] | 2010 | Brazil | Did not explicitly state | None stated | Multiple sites/population level | WHO |
| Tallapureddy et al.[168] | 2017 | India | Did not explicitly state | Up to 42 days postpartum | Tertiary/academic facility or equivalent | WHO |
| Tan et al.[169] | 2015 | China | Did not explicitly state | At least 20 weeks postpartum | Multiple sites/population level | Author defined |
| Tanimia et al.[170] | 2016 | Papua New Guinea | Antepartum, delivery, and postpartum | None stated | Tertiary/academic facility or equivalent | WHO-mod Haydom/Nelissen. |
| Thomas et al.[171] | 2005 | India | Antepartum and delivery | None stated | Tertiary/academic facility or equivalent | Author defined |
| Tuncalp et al.[172] | 2013 | Ghana | Did not explicitly state | Up to 42 days postpartum | Tertiary/academic facility or equivalent | WHO |
| Tuncalp et al.[173] | 2014 | Ghana | Did not explicitly state | None stated | Tertiary/academic facility or equivalent | WHO |
| Urquia et al.[174] | 2017 | Canada | Delivery | None stated | Multiple sites/population level | Joseph et al., Liu et al. |
| van den Akker et al.[175] | 2011 | Malawi | Did not explicitly state | Up to 42 days postpartum | Single site, level not specified | Author defined |
| Venkatesh et al.[176] | 2016 | India | Did not explicitly state | None stated | Single site, level not specified | WHO |
| Villar et al.[177] | 2007 | Latin America | Did not explicitly state | None stated | Multiple sites/population level | Author defined |
| Viteri et al.[178] | 2017 | United States | Did not explicitly state | None stated | Tertiary/academic facility or equivalent | Author defined |
| Wahlberg et al.[179] | 2013 | Sweden | Did not explicitly state | At least 28 weeks gestation | Multiple sites/population level | Author defined |
| Wandabwa et al.[180] | 2011 | Uganda | Did not explicitly state | None stated | Tertiary/academic facility or equivalent | Author defined |
| Wang et al.[181] | 2016 | United States | Did not explicitly state | At least 20 weeks gestation, only livebirths | Tertiary/academic facility or equivalent | Main et al. |
| Wanigaratne et al.[182] | 2015 | Canada | Delivery | None stated | Multiple sites/population level | CPSS |
| Waterstone et al.[14] | 2001 | England | Did not explicitly state | At least 24 weeks gestation | Multiple sites/population level | Author defined |
| Wen et al.[183] | 2005 | Canada | Did not explicitly state | None stated | Multiple sites/population level | Author defined |
| Yamamoto et al.[184] | 2018 | Japan | Did not explicitly state | At least 36 weeks gestation | Tertiary/academic facility or equivalent | Author defined |
| Yoong et al.[185] | 1996 | England | Antepartum, delivery, and postpartum | None stated | Single site, level not specified | Author defined |
| Young et al.[186] | 2018 | Canada | Did not explicitly state | None stated | Multiple sites/population level | Author defined |
| Zanardi et al.[187] | 2016 | Brazil | Did not explicitly state | None stated | Multiple sites/population level | WHO |
| Zanconato et al.[188] | 2012 | Italy | Did not explicitly state | Up to 42 days postpartum | Tertiary/academic facility or equivalent | Author defined |
| Zwart et al.[189] | 2008 | Netherlands | Did not explicitly state | Up to 42 days postpartum | Multiple sites/population level | Author defined |

**References**

19. Abha S, Chandrashekhar S, Sonal D. Maternal Near Miss: A Valuable Contribution in Maternal Care. J Obstet Gynaecol India. 2016;66(Suppl 1):217-22.

20. Adeoye IA, Onayade AA, Fatusi AO. Incidence, determinants and perinatal outcomes of near miss maternal morbidity in Ile-Ife Nigeria: A prospective case control study. BMC Pregnancy and Childbirth. 2013;13 [no pagination][93].

21. Almerie Y, Almerie MQ, Matar HE, Shahrour Y, Al Chamat AA, Abdulsalam A. Obstetric near-miss and maternal mortality in maternity university hospital, Damascus, Syria: a retrospective study. BMC Pregnancy Childbirth. 2010;10:65.

22. Amaral E, Souza JP, Surita F, Luz AG, Sousa MH, Cecatti JG, et al. A population-based surveillance study on severe acute maternal morbidity (near-miss) and adverse perinatal outcomes in Campinas, Brazil: The Vigimoma Project. BMC Pregnancy and Childbirth. 2011;11 [no pagination][9].

23. Angelini CR, Pacagnella RC, Parpinelli MA, Silveira C, Andreucci CB, Ferreira EC, et al. Post-Traumatic Stress Disorder and severe maternal morbidity: is there an association? Clinics. 2018;73:e309.

24. Assarag B, Dujardin B, Delamou A, Meski FZ, De Brouwere V. Determinants of maternal near-miss in morocco: Too late, too far, too sloppy? PloS one. 2015;10(1)[no pagination][e0116675].

25. Bakshi RK, Aggarwal P, Roy D, Nautiyal R, Kakkar R. Indicators of maternal 'near miss' morbidity at different levels of health care in North India: A pilot study. Bangladesh Journal of Medical Science. 2015;14(3):254-7.

26. Bakshi RK, Roy D, Aggarwal P, Nautiyal R, Chaturvedi J, Kakkar R. Application of WHO 'Near-Miss' tool indicates good quality of maternal care in rural healthcare setting in Uttarakhand, Northern India. Journal of Clinical and Diagnostic Research. 2016;10(1):LC10-LC3.

27. Bashour H, Saad-Haddad G, DeJong J, Ramadan MC, Hassan S, Breebaart M, et al. A cross sectional study of maternal 'near-miss' cases in major public hospitals in Egypt, Lebanon, Palestine and Syria. BMC Pregnancy and Childbirth. 2015;15(1)[no pagination][296].

28. Bateman BT, Mhyre JM, Hernandez-Diaz S, Huybrechts KF, Fischer MA, Creanga AA, et al. Development of a comorbidity index for use in obstetric patients. Obstetrics and gynecology. 2013;122(5):957-65.

29. Belanoff C, Declercq ER, Diop H, Gopal D, Kotelchuck M, Luke B, et al. Severe Maternal Morbidity and the Use of Assisted Reproductive Technology in Massachusetts. Obstetrics and gynecology. 2016;127(3):527-34.

30. Bolnga JW, Morris M, Totona C, Laman M. Maternal near-misses at a provincial hospital in Papua New Guinea: A prospective observational study. Aust N Z J Obstet Gynaecol. 2017;57(6):624-9.

31. Booker WA, Ananth CV, Wright JD, Siddiq Z, D'Alton ME, Cleary KL, et al. Trends in comorbidity, acuity, and maternal risk associated with preeclampsia across obstetric volume settings. J Matern Fetal Neonatal Med. 2018:1-8.

32. Bouvier-Colle MH, Mohangoo AD, Gissler M, Novak-Antolic Z, Vutuc C, Szamotulska K, et al. What about the mothers? An analysis of maternal mortality and morbidity in perinatal health surveillance systems in Europe. BJOG: An International Journal of Obstetrics and Gynaecology. 2012;119(7):880-9.

33. Brace V, Penney G, Hall M. Quantifying severe maternal morbidity: a Scottish population study. BJOG: An International Journal of Obstetrics & Gynaecology. 2004;111(5):481-4.

34. Brandt JS, Srinivas SK, Elovitz ME, Bastek JA. Does a maternal-fetal medicine-centered labor and delivery coverage model put the 'M' back in MFM? American Journal of Obstetrics & Gynecology. 2014;210(4):333.e1-.e7.

35. Brown HL, Small M, Taylor YJ, Chireau M, Howard DL. Near Miss Maternal Mortality in a Multiethnic Population. Ann Epidemiol. 2011;21(2):73-7.

36. Callaghan WM, Mackay AP, Berg CJ. Identification of severe maternal morbidity during delivery hospitalizations, United States, 1991-2003. American Journal of Obstetrics & Gynecology. 2008;199(2):133.e1-8.

37. Calvo-Aguilar O, Morales-Garcia VE, Fabian-Fabian J. Extreme maternal morbidity in the Hospital General Dr. Aurelio Valdivieso, Oaxaca Health Services. Ginecologia y Obstetricia de Mexico. 2010;78(12):660-8.

38. Camargo RS, Pacagnella RC, Cecatti JG, Parpinelli MA, Souza JP, Sousa MH. Subsequent reproductive outcome in women who have experienced a potentially life-threatening condition or a maternal near-miss during pregnancy. Clinics. 2011;66(8):1367-72.

39. Cecatti JG, Souza JP, Oliveira Neto AF, Parpinelli MA, Sousa MH, Say L, et al. Pre-validation of the WHO organ dysfunction based criteria for identification of maternal near miss. Reprod Health. 2011;8(1)[no pagination][22].

40. Cecatti JG, Souza RT, Pacagnella RC, Leal MC, Moura EC, Santos LM. Maternal near miss among women using the public health system in the Amazon and Northeast regions of Brazil. Rev Panam Salud Publica. 2015;37(4-5):232-8.

41. Cham M, Sundby J, Siri V. Fetal outcome in severe maternal morbidity: Too many stillbirths. International Journal of Gynecology and Obstetrics. 2009;2:S138.

42. Chantry AA, Deneux-Tharaux C, Cans C, Ego A, Quantin C, Bouvier-Colle MH. Hospital discharge data can be used for monitoring procedures and intensive care related to severe maternal morbidity. J Clin Epidemiol. 2011;64[9]:1014-22.

43. Chaudhuri S, Nath S. Life-threatening Complications in Pregnancy in a Teaching Hospital in Kolkata, India. Journal of Obstetrics and Gynecology of India. 2018:1-8.

44. Chhabra P, Guleria K, Saini NK, Anjur KT, Vaid NB. Pattern of severe maternal morbidity in a tertiary hospital of Delhi, India: a pilot study. Trop Doct. 2008;38(4):201-4.

45. Colmorn LB, Petersen KB, Jakobsson M, Lindqvist PG, Klungsoyr K, Kallen K, et al. The Nordic Obstetric Surveillance Study: A study of complete uterine rupture, abnormally invasive placenta, peripartum hysterectomy, and severe blood loss at delivery. Acta Obstetricia et Gynecologica Scandinavica. 2015;94(7):734-44.

46. Creanga AA, Bateman BT, Kuklina EV, Callaghan WM. Racial and ethnic disparities in severe maternal morbidity: a multistate analysis, 2008-2010. American Journal of Obstetrics & Gynecology. 2014;210(5):435.e1-8.

47. Cromi A, Marconi N, Casarin J, Cominotti S, Pinelli C, Riccardi M, et al. Maternal intra and postpartum near-miss following assisted reproductive technology: a retrospective study. BJOG: An International Journal of Obstetrics & Gynaecology. 2018;05:05.

48. Das I, Datta M, Samanta S, Mahapatra B, Mukherjee P. A cross-sectional study on post-partum severe acute maternal morbidity and maternal deaths in a tertiary level teaching hospital of Eastern India. International Journal of Women's Health and Reproduction Sciences. 2014;2(3):113-8.

49. David E, Machungo F, Zanconato G, Cavaliere E, Fiosse S, Sululu C, et al. Maternal near miss and maternal deaths in Mozambique: a cross-sectional, region-wide study of 635 consecutive cases assisted in health facilities of Maputo province. BMC Pregnancy Childbirth. 2014;14:401.

50. De Mucio B, Abalos E, Cuesta C, Carroli G, Serruya S, Giordano D, et al. Maternal near miss and predictive ability of potentially life-threatening conditions at selected maternity hospitals in Latin America. Reprod Health. 2016;13(1):1-10.

51. De Souza MA, De Souza TH, Goncalves AK. Determinants of maternal near miss in an obstetric intensive care unit. Revista Brasileira de Ginecologia e Obstetricia. 2015; 37(11):498-504

52. Dias MA, Domingues RM, Schilithz AO, Nakamura-Pereira M, Diniz CS, Brum IR, et al. Incidence of maternal near miss in hospital childbirth and postpartum: data from the Birth in Brazil study. Cad Saude Publica. 2014;30:S1-S12.

53. El Ghardallou M, Ajmi TN, Mkhazni A, Zedini C, Meddeb S, Khairi H, et al. Maternal Near Miss and Quality of Obstetric Care in a Tunisian Tertiary Level Maternity. Afr J Reprod Health. 2016;20[4]:44-50.

54. Farchi S, Polo A, Franco F, Di Lallo D, Guasticchi G. Severe postpartum morbidity and mode of delivery: a retrospective cohort study. Acta Obstetricia et Gynecologica Scandinavica. 2010;89(12)1600-3.

55. Frederiksen B, Lillehoj C, Kane D, Goodman D, Rankin K. Evaluating Iowa Severe Maternal Morbidity Trends and Maternal Risk Factors: 2009-2014. Matern Child Health J. 2017;21(9):1834-44.

56. Friedman AM, Ananth CV, Huang Y, D'Alton ME, Wright JD. Hospital delivery volume, severe obstetrical morbidity, and failure to rescue. American Journal of Obstetrics & Gynecology. 2016;215(6):795.e1-.e14.

57. Furuta M, Sandall J, Cooper D, Bick D. The relationship between severe maternal morbidity and psychological health symptoms at 6-8 weeks postpartum: a prospective cohort study in one English maternity unit. BMC Pregnancy Childbirth. 2014;14:133.

58. Galindo Mateu N, Roig Casaban N, Moreno Collado A, Gurrea Soteras M, Alberola Cunat V, Diago Almela V, et al. Near-miss in a referral hospital. [Spanish]. Progresos de Obstetricia y Ginecologia.

59. Galvao LP, Alvim-Pereira F, de Mendonca CM, Menezes FE, Gois KA, Ribeiro RF, Jr., et al. The prevalence of severe maternal morbidity and near miss and associated factors in Sergipe, Northeast Brazil. BMC Pregnancy Childbirth. 2014;14:25.

60. Gandhi MN, Welz T, Ronsmans C. Severe acute maternal morbidity in rural South Africa. Int J Gynaecol Obstet. 2004;87[2]:180-7.

61. Gebrehiwot Y, Tewolde BT. Improving maternity care in Ethiopia through facility based review of maternal deaths and near misses. International Journal of Gynecology and Obstetrics. 2014;127[S1]:S29-S34.

62. Geller SE, Rosenberg D, Cox SM, Kilpatrick S. Defining a conceptual framework for near-miss maternal morbidity. J Am Med Womens Assoc. 2002;57[3]:135-9.

63. Ghazal-Aswad S, Badrinath P, Sidky I, Safi TH, Gargash H, Abdul-Razak Y, et al. Severe acute maternal morbidity in a high-income developing multiethnic country. Matern Child Health J. 2013;17[3]:399-404.

64. Ghazivakili Z, Lotfi R, Kabir K, nia RN, Naeeni MR. Maternal near miss approach to evaluate quality of care in Alborz province, Iran. Midwifery. 2016;41:118-24.

65. Gibson C, Rohan AM, Gillespie KH. Severe Maternal Morbidity During Delivery Hospitalizations. Wmj. 2017;116[5]:215-20.

66. Girard F, Burlet G, Bayoumeu F, Fresson J, Bouvier-Colle MH, Boutroy JL. [Severe complications of pregnancy and delivery: the situation in Lorraine based on the European investigation]. J Gynecol Obstet Biol Reprod [Paris]. 2001;30[6 Suppl]:S10-7.

67. Goldenberg RL, Saleem S, Ali S, Moore JL, Lokangako A, Tshefu A, et al. Maternal near miss in low-resource areas. Int J Gynaecol Obstet. 2017;138(30):347-55.

68. Haddad SM, Cecatti JG, Parpinelli MA, Souza JP, Costa ML, Sousa MH, et al. From planning to practice: building the national network for the Surveillance of Severe Maternal Morbidity. BMC Public Health. 2011;11:283.

69. Hassan SJ, Wick L, DeJong J. A glance into the hidden burden of maternal morbidity and patterns of management in a Palestinian governmental referral hospital. Women Birth. 2015;28(4):e148-56.

70. Herklots T, van Acht L, Meguid T, Franx A, Jacod B. Severe maternal morbidity in Zanzibar's referral hospital: Measuring the impact of in-hospital care. PLoS ONE [Electronic Resource]. 2017;12(8):e0181470.

71. Howell EA, Egorova N, Balbierz A, Zeitlin J, Hebert PL. Black-white differences in severe maternal morbidity and site of care. American Journal of Obstetrics & Gynecology. 2016;214(1):122.e1-7.

72. Howell EA, Egorova NN, Balbierz A, Zeitlin J, Hebert PL. Site of delivery contribution to black-white severe maternal morbidity disparity. American Journal of Obstetrics & Gynecology. 2016;215(2):143-52.

73. Howell EA, Zeitlin J, Hebert PL, Balbierz A, Egorova N. Association between hospital-level obstetric quality indicators and maternal and neonatal morbidity. Jama. 2014;312(15):1531-41.

74. Iwuh IA, Fawcus S, Schoeman L. Maternal near-miss audit in the metro west maternity service, Cape Town, South Africa: A retrospective observational study. South African Medical Journal. 2018;108(3):171-5.

75. Jabir M, Abdul-Salam I, Suheil DM, Al-Hilli W, Abul-Hassan S, Al-Zuheiri A, et al. Maternal near miss and quality of maternal health care in Baghdad, Iraq. BMC Pregnancy Childbirth. 2013;13:11.

76. Jayaratnam S, Burton A, Connan KF, de Costa C. Maternal 'near miss' at Royal Darwin Hospital: An analysis of severe maternal morbidity at an Australian regional tertiary maternity unit. Aust N Z J Obstet Gynaecol. 2016;56(4):381-6.

77. Jayaratnam S, De Costa C, Howat P. Developing an assessment tool for maternal morbidity 'near-miss'- a prospective study in a large Australian regional hospital. Aust N Z J Obstet Gynaecol. 2011;51(5):421-5.

78. Kalisa R, Rulisa S, van den Akker T, van Roosmalen J. Maternal Near Miss and quality of care in a rural Rwandan hospital. BMC Pregnancy Childbirth. 2016;16(1):324.

79. Kiruja J, Osman F, Egal JA, Essen B, Klingberg-Allvin M, Erlandsson K. Maternal near-miss and death incidences - Frequencies, causes and the referral chain in Somaliland: A pilot study using the WHO near-miss approach. Sex Reprod Healthc. 2017;(12):30-6.

80. Knight M, Kurinczuk JJ, Spark P, Brocklehurst P. Inequalities in maternal health: national cohort study of ethnic variation in severe maternal morbidities. BMJ (Clinical research ed). 2009;338:b542.

81. Kulkarni R, Chauhan S, Daver R, Nandanwar Y, Patil A, Bhosale A. Prospective observational study of near-miss obstetric events at two tertiary hospitals in Mumbai, Maharashtra, India. Int J Gynaecol Obstet. 2016;132(2):170-3.

82. Lazariu V, Nguyen T, McNutt LA, Jeffrey J, Kacica M. Severe maternal morbidity: A population-based study of an expanded measure and associated factors. PLoS ONE (Electronic Resource). 2017;12(8):e0182343.

83. Letois F, Morau E, Pastor J, Pellecuer D, Mercier G. Cost of severe maternal morbidity in 2011 in Languedoc-Roussillon, France. Revue d'Epidemiologie et de Sante Publique. 2016;64(5):359-66.

84. Linard M, Blondel B, Estellat C, Deneux-Tharaux C, Luton D, Oury JF, et al. Association between inadequate antenatal care utilisation and severe perinatal and maternal morbidity: an analysis in the PreCARE cohort. BJOG: An International Journal of Obstetrics and Gynaecology. 2018;125(5):587-95.

85. Lindquist A, Knight M, Kurinczuk JJ. Variation in severe maternal morbidity according to socioeconomic position: a UK national case-control study. BMJ Open. 2013;3(6):20.

86. Lindquist A, Noor N, Sullivan E, Knight M. The impact of socioeconomic position on severe maternal morbidity outcomes among women in Australia: a national case-control study. BJOG: An International Journal of Obstetrics & Gynaecology. 2015;122(12):1601-9.

87. Lindquist AC, Kurinczuk JJ, Wallace EM, Oats J, Knight M. Risk factors for maternal morbidity in Victoria, Australia: a population-based study. BMJ Open. 2015;5(8):e007903.

88. Lisonkova S, Muraca GM, Potts J, Liauw J, Chan WS, Skoll A, et al. Association Between Prepregnancy Body Mass Index and Severe Maternal Morbidity. Jama. 2017;318(18):1777-86.

89. Lisonkova S, Potts J, Muraca GM, Razaz N, Sabr Y, Chan WS, et al. Maternal age and severe maternal morbidity: A population-based retrospective cohort study. PLoS Med. 2017;14(5):e1002307.

90. Litorp H, Roost M, Kidanto HL, Nystrom L, Essen B. The effects of previous cesarean deliveries on severe maternal and adverse perinatal outcomes at a university hospital in Tanzania. Int J Gynaecol Obstet. 2016;133(2):183-7.

91. Liyew EF, Yalew AW, Afework MF, Essen B. Incidence and causes of maternal near-miss in selected hospitals of Addis Ababa, Ethiopia. PLoS ONE (Electronic Resource). 2017;12(6):e0179013.

92. Lori JR, Starke AE. A critical analysis of maternal morbidity and mortality in Liberia, West Africa. Midwifery. 2012;28(1):67-72.

93. Lotufo FA, Parpinelli MA, Haddad SM, Surita FG, Cecatti JG. Applying the new concept of maternal near-miss in an intensive care unit. Clinics. 2012;67(3):225-30.

94. Lutomski JE, Byrne BM, Greene RA. Severe maternal morbidity in Ireland: What are the rates? what are the risks? Archives of Disease in Childhood: Fetal and Neonatal Edition. 2012;1:A39.

95. Luz AG, Tiago DB, Da Silva JCG, Amaral E. Severe maternal morbidity at a local reference university hospital in Campinas, Sao Paulo, Brazil. [Portuguese]. Rev. 2008;30(6):281-6.

96. Lynch CM, Sheridan C, Breathnach FM, Said S, Daly S, Bryne B. Near miss maternal morbidity. Ir Med J. 2008;101(5).

97. Madeiro AP, Rufino AC, Lacerda e Z, Brasil LG. Incidence and determinants of severe maternal morbidity: A transversal study in a referral hospital in Teresina, Piaui, Brazil. BMC Pregnancy and Childbirth. 2015;15(1)[no pagination][210].

98. Magalhaes MC, Bustamante-Teixeira MT. Severe acute maternal morbidity: Use of the Brazilian hospital information system. Rev Saude Publica. 2012;46(3):472-8.

99. Main EK, Abreo A, McNulty J, Gilbert W, McNally C, Poeltler D, et al. Measuring severe maternal morbidity: validation of potential measures. American Journal of Obstetrics & Gynecology. 2016;214(5):643.e1-.e10.

100. Main EK, Cape V, Abreo A, Vasher J, Woods A, Carpenter A, et al. Reduction of severe maternal morbidity from hemorrhage using a state perinatal quality collaborative. American Journal of Obstetrics & Gynecology. 2017;216(3):298.e1-.e11.

101. Marcellin L, Delorme P, Bonnet MP, Grange G, Kayem G, Tsatsaris V, et al. Placenta percreta is associated with more frequent severe maternal morbidity than placenta accreta. American Journal of Obstetrics & Gynecology. 2018;04:04.

102. Martin AS, Monsour M, Kissin DM, Jamieson DJ, Callaghan WM, Boulet SL. Trends in Severe Maternal Morbidity After Assisted Reproductive Technology in the United States, 2008-2012. Obstetrics & Gynecology. 2016;127(1):59-66.

103. Mawarti Y, Utarini A, Hakimi M. Maternal care quality in near miss and maternal mortality in an academic public tertiary hospital in Yogyakarta, Indonesia: a retrospective cohort study. BMC Pregnancy Childbirth. 2017;17(1):149.

104. Mbachu, II, Ezeama C, Osuagwu K, Umeononihu OS, Obiannika C, Ezeama N. A cross sectional study of maternal near miss and mortality at a rural tertiary centre in southern nigeria. BMC Pregnancy Childbirth. 2017;17(1):251.

105. Mekango DE, Alemayehu M, Gebregergs GB, Medhanyie AA, Goba G. Determinants of maternal near miss among women in public hospital maternity wards in Northern Ethiopia: A facility based case-control study. PloS one. 2017;12(9)[no pagination][e0183886].

106. Metcalfe A, Wick J, Ronksley P. Racial disparities in comorbidity and severe maternal morbidity/mortality in the United States: an analysis of temporal trends. Acta Obstetricia et Gynecologica Scandinavica. 2018;97(1):89-96.

107. Mohammadi S, Essen B, Fallahian M, Taheripanah R, Saleh Gargari S, Kallestal C. Maternal near-miss at university hospitals with cesarean overuse: an incident case-control study. Acta Obstet Gynecol Scand. 2016;95(7):777-86.

108. Mohammadi S, Saleh Gargari S, Fallahian M, Kallestal C, Ziaei S, Essen B. Afghan migrants face more suboptimal care than natives: a maternal near-miss audit study at university hospitals in Tehran, Iran. BMC Pregnancy Childbirth. 2017;17(1):64.

109. Moraes AP, Barreto SM, Passos VM, Golino PS, Costa JA, Vasconcelos MX. Incidence and main causes of severe maternal morbidity in Sao Luis, Maranhao, Brazil: a longitudinal study. Sao Paulo Med J. 2011;129(3):146-52.

110. Muraca GM, Sabr Y, Lisonkova S, Skoll A, Brant R, Cundiff GW, et al. Perinatal and maternal morbidity and mortality after attempted operative vaginal delivery at midpelvic station. CMAJ Canadian Medical Association Journal. 2017;189(22):E764-E72.

111. Muraca GM, Skoll A, Lisonkova S, Sabr Y, Brant R, Cundiff GW, et al. Perinatal and maternal morbidity and mortality among term singletons following midcavity operative vaginal delivery versus caesarean delivery. BJOG: An International Journal of Obstetrics & Gynaecology. 2018;125(6):693-702.

112. Murphy CM, Murad K, Deane R, Byrne B, Geary MP, McAuliffe FM. Severe maternal morbidity for 2004-2005 in the three Dublin maternity hospitals. Eur J Obstet Gynecol Reprod Biol. 2009;143(1):34-7.

113. Naderi T, Foroodnia S, Omidi S, Samadani F, Nakhaee N. Incidence and correlates of maternal near miss in southeast iran. int. 2015;2015:914713.

114. Nair M, Kurinczuk JJ, Knight M. Ethnic variations in severe maternal morbidity in the UK- a case control study. PLoS ONE [Electronic Resource]. 2014;9(4):e95086.

115. Nair M, Kurinczuk JJ, Knight M. Establishing a national maternal morbidity outcome indicator in England: A population- based study using routine hospital data. PloS one. 2016;11(4)[no pagination][e0153370].

116. Nakamura-Pereira M, Mendes-Silva W, Dias MA, Reichenheim ME, Lobato G. [The Hospital Information System of the Brazilian Unified National Health System: a performance evaluation for auditing maternal near miss]. Cad Saude Publica. 2013;29(7):1333-45.

117. Nakimuli A, Mbalinda SN, Nabirye RC, Kakaire O, Nakubulwa S, Osinde MO, et al. Still births, neonatal deaths and neonatal near miss cases attributable to severe obstetric complications: a prospective cohort study in two referral hospitals in Uganda. BMC Pediatr. 2015;15:44.

118. Nakimuli A, Nakubulwa S, Kakaire O, Osinde MO, Mbalinda SN, Kakande N, et al. The burden of maternal morbidity and mortality attributable to hypertensive disorders in pregnancy: a prospective cohort study from Uganda. BMC Pregnancy Childbirth. 2016;16:205.

119. Nakimuli A, Nakubulwa S, Kakaire O, Osinde MO, Mbalinda SN, Nabirye RC, et al. Maternal near misses from two referral hospitals in Uganda: a prospective cohort study on incidence, determinants and prognostic factors. BMC Pregnancy Childbirth. 2016;16:24.

120. Nansubuga E, Ayiga N, Moyer CA. Prevalence of maternal near miss and community-based risk factors in Central Uganda. Int J Gynaecol Obstet. 2016;135(2):214-20.

121. Nelissen EJ, Mduma E, Ersdal HL, Evjen-Olsen B, van Roosmalen JJ, Stekelenburg J. Maternal near miss and mortality in a rural referral hospital in northern Tanzania: a cross-sectional study. BMC Pregnancy Childbirth. 2013;13:141.

122. Norhayati MN, Nik Hazlina NH, Aniza AA, Asrenee AR. Severe Maternal Morbidity and Postpartum Depressive Symptomatology: A Prospective Double Cohort Comparison Study. Res Nurs Health. 2016;39(6):415-25.

123. Norhayati MN, Nik Hazlina NH, Sulaiman Z, Azman MY. Severe maternal morbidity and near misses in tertiary hospitals, Kelantan, Malaysia: a cross-sectional study. BMC Public Health. 2016;16:229.

124. Norhayati MN, Hazlina NHN, Asrenee AR, Sulaiman Z. The experiences of women with maternal near miss and their perception of quality of care in Kelantan, Malaysia: A qualitative study. BMC Pregnancy and Childbirth. 2017;17(1)[no pagination][189].

125. Norhayati MN, Nik Hazlina NH, Aniza AA. Functional status of women with and without severe maternal morbidity: A prospective cohort study. Women Birth. 2016;29(5):443-9.

126. O'Malley EG, Popivanov P, Fergus A, Tan T, Byrne B. Maternal near miss: what lies beneath? Eur J Obstet Gynecol Reprod Biol. 2016;199:116-20.

127. Olagbuji BN, Ezeanochie MC, Igbaruma S, Okoigi SO, Ande AB. Stillbirth in cases of severe acute maternal morbidity. Int J Gynaecol Obstet. 2012;119(1):53-6.

128. Oliveira LC, Costa AARD. Fetal and neonatal deaths among cases of maternal near miss. [Portuguese]. Rev Assoc Med Bras. 2013;59(5):487-94.

129. Ozimek JA, Eddins RM, Greene N, Karagyozyan D, Pak S, Wong M, et al. Opportunities for improvement in care among women with severe maternal morbidity. American Journal of Obstetrics & Gynecology. 2016;215(4):509.e1-6.

130. Pacheco AJ, Katz L, Souza AS, de Amorim MM. Factors associated with severe maternal morbidity and near miss in the Sao Francisco Valley, Brazil: a retrospective, cohort study. BMC Pregnancy Childbirth. 2014;14:91.

131. Pallasmaa N, Ekblad U, Gissler M. Severe maternal morbidity and the mode of delivery. Acta Obstet Gynecol Scand. 2008;87(6):662-8.

132. Pallasmaa N, Ekblad U, Gissler M, Alanen A. The impact of maternal obesity, age, pre-eclampsia and insulin dependent diabetes on severe maternal morbidity by mode of delivery-a register-based cohort study. Arch Gynecol Obstet. 2015;291(2):311-8.

133. Panday M, Mantel GD, Moodley J. Audit of severe acute morbidity in hypertensive pregnancies in a developing country. J Obstet Gynaecol. 2004;24(4):387-91.

134. Pandey A, Das V, Agarwal A, Agrawal S, Misra D, Jaiswal N. Evaluation of obstetric near miss and maternal deaths in a tertiary care hospital in north India: shifting focus from mortality to morbidity. J Obstet Gynaecol India. 2014;64(6):394-9.

135. Parmar NT, Parmar AG, Mazumdar VS. Incidence of Maternal "Near-Miss" Events in a Tertiary Care Hospital of Central Gujarat, India. J Obstet Gynaecol India. 2016;66(Suppl 1):315-20.

136. Pattinson RC, Buchmann E, Mantel G, Schoon M, Rees H. Can enquiries into severe acute maternal morbidity act as a surrogate for maternal death enquiries? BJOG: An International Journal of Obstetrics & Gynaecology. 2003;110(10):889-93.

137. Prual A, Huguet D, Garbin O, Rabe G. Severe obstetric morbidity of the third trimester, delivery and early puerperium in Niamey [Niger]. Afr J Reprod Health. 1998;2(1):10-9.

138. Prual A, Bouvier-Colle MH, de Bernis L, Breart G. Severe maternal morbidity from direct obstetric causes in West Africa: incidence and case fatality rates. Bulletin of the World Health Organization. 2000;78(5):593-602.

139. Ps R, Verma S, Rai L, Kumar P, Pai MV, Shetty J. "Near miss" obstetric events and maternal deaths in a tertiary care hospital: an audit. J Pregnancy. 2013;2013:393758.

140. Rabia S, Tabbasum R, Perveen F, Ali A, Reeta. Pattern and obstetric risk factors of severe acute maternal morbidity (samm) and maternal death in tertiary care hospital karachi. Medical Channel. 2011;17(1):28-33.

141. Rana A, Baral G, Dangal G. Maternal near-miss: a multicenter surveillance in Kathmandu Valley. Jnm. 2013;52(190):299-304.

142. Rathod AD, Chavan RP, Bhagat V, Pajai S, Padmawar A, Thool P. Analysis of near-miss and maternal mortality at tertiary referral centre of rural India. J Obstet Gynaecol India. 2016;66(Suppl 1):295-300.

143. Reena RP, Radha KR. Factors associated with maternal near miss: A study from Kerala. Indian J Public Health. 2018;62(1):58-60.

144. Reid LD, Creanga AA. Severe maternal morbidity and related hospital quality measures in Maryland. J Perinatol. 2018;28:28.

145. Reime B, Janssen PA, Farris L, Borde T, Hellmers C, Myezwa H, et al. Maternal near-miss among women with a migrant background in Germany. Acta Obstet Gynecol Scand. 2012;91(7):824-9.

146. Roberts CL, Ford JB, Algert CS, Bell JC, Simpson JM, Morris JM. Trends in adverse maternal outcomes during childbirth: a population-based study of severe maternal morbidity. BMC Pregnancy Childbirth. 2009;9:7.

147. Ronsmans C, Cresswell JA, Goufodji S, Agbla S, Ganaba R, Assarag B, et al. Characteristics of neonatal near miss in hospitals in Benin, Burkina Faso and Morocco in 2012-2013. Trop Med Int Health. 2016;21(4):535-45.

148. Roost M, Altamirano VC, Liljestrand J, Essen B. Priorities in emergency obstetric care in Bolivia--maternal mortality and near-miss morbidity in metropolitan La Paz. BJOG: An International Journal of Obstetrics & Gynaecology. 2009;116(9):1210-7.

149. Rosendo TM, Roncalli AG. Prevalence and factors associated with Maternal Near Misses: a survey of the population in a capital city of the Brazilian Northeast. [Portuguese]. Cienc. 2015;20(4):1295-304.

150. Rulisa S, Umuziranenge I, Small M, van Roosmalen J. Maternal near miss and mortality in a tertiary care hospital in Rwanda. BMC Pregnancy Childbirth. 2015;15:203.

151. Sahijwani DV, Desai A, Kansara V. Analysis of near miss cases as a reflection of emergency obstetric services and need of obstetric ICCU. Journal of SAFOG. 2013;5(3):99-101.

152. Sangeeta G, Leena W, Taru G, Sushma K, Nupur G, Amrita P. Evaluation of severe maternal outcomes to assess quality of maternal health care at a tertiary center. J Obstet Gynaecol India. 2015;65(1):23-7.

153. Sayinzoga F, Bijlmakers L, van der Velden K, van Dillen J. Severe maternal outcomes and quality of care at district hospitals in Rwanda- a multicentre prospective case-control study. BMC Pregnancy Childbirth. 2017;17(1):394.

154. Serruya SJ, de Mucio B, Martinez G, Mainero L, de Francisco A, Say L, et al. Exploring the Concept of Degrees of Maternal Morbidity as a Tool for Surveillance of Maternal Health in Latin American and Caribbean Settings. BioMed Research International. 2017;2017:8271042.

155. Shields LE, Wiesner S, Klein C, Pelletreau B, Hedriana HL. Use of Maternal Early Warning Trigger tool reduces maternal morbidity. American Journal of Obstetrics & Gynecology. 2016;214(4):527.e1-.e6.

156. Shields LE, Wiesner S, Klein C, Pelletreau B, Hedriana HL. Early standardized treatment of critical blood pressure elevations is associated with a reduction in eclampsia and severe maternal morbidity. American Journal of Obstetrics & Gynecology. 2017;216(4):415.e1-.e5.

157. Shrestha NS, Saha R, Karki C. Near miss maternal morbidity and maternal mortality at Kathmandu Medical College Teaching Hospital. Kathmandu Univ. 2010;8(30):222-6.

158. Siddiqui SA, Soomro N, Shabih-ul-Hasnain F. Severe obstetric morbidity and its outcome in patients presenting in a tertiary care hospital of Karachi. JPMA J Pak Med Assoc. 2012;62(3):226-31.

159. Sigakis MJ, Leffert LR, Mirzakhani H, Sharawi N, Rajala B, Callaghan WM, et al. The Validity of Discharge Billing Codes Reflecting Severe Maternal Morbidity. Anesth Analg. 2016;123(3):731-8.

160. Sikder SS, Labrique AB, Shamim AA, Ali H, Mehra S, Wu L, et al. Risk factors for reported obstetric complications and near misses in rural northwest Bangladesh: analysis from a prospective cohort study. BMC Pregnancy Childbirth. 2014;14:347.

161. Silva TC, Varela PL, Oliveira RR, Mathias TA. Severe maternal morbidity identified in the Hospital Information System of the Brazilian National Health System in Parana State, Brazil, 2010. Epidemiol. 2016;25(3):617-28.

162. Silveira C, Souza RT, Costa ML, Parpinelli MA, Pacagnella RC, Ferreira EC, et al. Validation of the WHO Disability Assessment Schedule [WHODAS 2.0] 12-item tool against the 36-item version for measuring functioning and disability associated with pregnancy and history of severe maternal morbidity. Int J Gynaecol Obstet. 2018;141 Suppl 1:39-47.

163. Soma-Pillay P, Pattinson RC, Langa-Mlambo L, Nkosi BS, Macdonald AP. Maternal near miss and maternal death in the Pretoria Academic Complex, South Africa: A population-based study. Samj, S. 2015;105(7):578-63.

164. Souza JP, Gulmezoglu AM, Vogel J, Carroli G, Lumbiganon P, Qureshi Z, et al. Moving beyond essential interventions for reduction of maternal mortality [the WHO Multicountry Survey on Maternal and Newborn Health]: a cross-sectional study. Lancet [London, England]. 2013;381(9879):1747-55.

165. Souza JP, Cecatti JG, Pacagnella RC, Giavarotti TM, Parpinelli MA, Camargo RS, et al. Development and validation of a questionnaire to identify severe maternal morbidity in epidemiological surveys. Reprod Health. 2010;7:16.

166. Souza JP, Cecatti JG, Parpinelli MA, Serruya SJ, Amaral E. Appropriate criteria for identification of near-miss maternal morbidity in tertiary care facilities: a cross sectional study. BMC Pregnancy Childbirth. 2007;7:20.

167. Souza JP, Cecatti JG, Parpinelli MA, Sousa MH, Lago TG, Pacagnella RC, et al. Maternal morbidity and near miss in the community: findings from the 2006 Brazilian demographic health survey. BJOG: An International Journal of Obstetrics & Gynaecology. 2010;117(13):1586-92.

168. Tallapureddy S, Velagaleti R, Palutla H, Satti CV. "Near-Miss" Obstetric events and maternal mortality in a Tertiary Care Hospital. Indian J Public Health. 2017;61(4):305-8.

169. Tan J, Liu XH, Yu C, Chen M, Chen XF, Sun X, et al. Effects of medical co-morbidities on severe maternal morbidities in China: a multicenter clinic register study. Acta Obstet Gynecol Scand. 2015;94(8):861-8.

170. Tanimia H, Jayaratnam S, Mola GL, Amoa AB, Costa C. Near-misses at the Port Moresby General Hospital: a descriptive study. Aust N Z J Obstet Gynaecol. 2016;56(2):148-53.

171. Thomas T, Jophy R, Mhaskar A, Misquith D. Are we increasing serious maternal morbidity by postponing termination of pregnancy in severe pre-eclampsia/eclampsia? J Obstet Gynaecol. 2005;25(4):347-51.

172. Tuncalp O, Hindin MJ, Adu-Bonsaffoh K, Adanu RM. Assessment of maternal near-miss and quality of care in a hospital-based study in Accra, Ghana. Int J Gynaecol Obstet. 2013;123(1):58-63.

173. Tuncalp O, Hindin MJ, Adu-Bonsaffoh K, Adanu RM. Understanding the continuum of maternal morbidity in Accra, Ghana. Maternal and child health journal. 2014;18(7):1648-57.

174. Urquia ML, Wanigaratne S, Ray JG, Joseph KS. Severe Maternal Morbidity Associated With Maternal Birthplace: A Population-Based Register Study. Journal of Obstetrics & Gynaecology Canada: JOGC. 2017;39(11):978-87.

175. van den Akker T, van Rhenen J, Mwagomba B, Lommerse K, Vinkhumbo S, van Roosmalen J. Reduction of severe acute maternal morbidity and maternal mortality in Thyolo District, Malawi: the impact of obstetric audit. PLoS ONE [Electronic Resource]. 2011;6(6):e20776.

176. Venkatesh S, Chinmayi, Ramkumar V, Sheela CN, Thomas A. Implementation of WHO Near-Miss Approach for Maternal Health at a Tertiary Care Hospital: An Audit. J Obstet Gynaecol India. 2016;66(4):259-62.

177. Villar J, Carroli G, Zavaleta N, Donner A, Wojdyla D, Faundes A, et al. Maternal and neonatal individual risks and benefits associated with caesarean delivery: Multicentre prospective study. Br Med J. 2007;335(7628):1025-9.

178. Viteri OA, England JA, Alrais MA, Lash KA, Villegas MI, Balogun OAA, et al. Association of nonsteroidal antiinflammatory drugs and postpartum hypertension in women with preeclampsia with severe features. Obstetrics and gynecology. 2017;130(4):830-5.

179. Wahlberg A, Roost M, Haglund B, Hogberg U, Essen B. Increased risk of severe maternal morbidity (near-miss) among immigrant women in Sweden: a population register-based study. BJOG: An International Journal of Obstetrics & Gynaecology. 2013;120(13):1605-11; discussion 12.

180. Wandabwa JN, Doyle P, Longo-Mbenza B, Kiondo P, Khainza B, Othieno E, et al. Human immunodeficiency virus and AIDS and other important predictors of maternal mortality in Mulago Hospital Complex Kampala Uganda. BMC Public Health. 2011;11:565.

181. Wang ET, Ozimek JA, Greene N, Ramos L, Vyas N, Kilpatrick SJ, et al. Impact of fertility treatment on severe maternal morbidity. Fertil Steril. 2016;106(2):423-6.

182. Wanigaratne S, Cole DC, Bassil K, Hyman I, Moineddin R, Urquia ML. Contribution of HIV to Maternal Morbidity Among Refugee Women in Canada. Am J Public Health. 2015;105(12):2449-56.

183. Wen SW, Huang L, Liston R, Heaman M, Baskett T, Rusen ID, et al. Severe maternal morbidity in Canada, 1991-2001. Cmaj. 2005;173(7):759-63.

184. Yamamoto R, Ishii K, Muto H, Ota S, Kawaguchi H, Hayashi S, et al. Incidence of and risk factors for severe maternal complications associated with hypertensive disorders after 36weeks' gestation in uncomplicated twin pregnancies: A prospective cohort study. J Obstet Gynaecol Res. 2018;19:19.

185. Yoong A, Nunns D, Raychaudhuri K. The use of near-miss maternal morbidity to improve the quality of obstetric care. Contemporary Reviews in Obstetrics and Gynaecology. 1996;8:143-6

186. Young CB, Liu S, Muraca GM, Sabr Y, Pressey T, Liston RM, et al. Mode of delivery after a previous cesarean birth, and associated maternal and neonatal morbidity. CMAJ Canadian Medical Association Journal. 2018;190(18):E556-E64.

187. Zanardi DM, Moura EC, Santos LP, Leal MC, Cecatti JG. The effect of maternal near miss on adverse infant nutritional outcomes. Clinics. 2016;71(10):593-9.

188. Zanconato G, Cavaliere E, Iacovella C, Vassanelli A, Schweiger V, Cipriani S, et al. Severe maternal morbidity in a tertiary care centre of northern Italy: a 5-year review. J Matern Fetal Neonatal Med. 2012;25(7):1025-8.

189. Zwart JJ, Richters JM, Ory F, de Vries JI, Bloemenkamp KW, van Roosmalen J. Severe maternal morbidity during pregnancy, delivery and puerperium in the Netherlands: a nationwide population-based study of 371,000 pregnancies.[Reprint in Ned Tijdschr Geneeskd. 2009 Apr 11;153(15):691-7; PMID: 19452769]. BJOG: An International Journal of Obstetrics & Gynaecology. 2008;115(7):842-50.
